# *This* shoe, *that* tiger: Semantic properties reflecting manual affordances of the referent modulate demonstrative use

**DOI:** 10.1371/journal.pone.0210333

**Published:** 2019-01-07

**Authors:** Roberta Rocca, Kristian Tylén, Mikkel Wallentin

**Affiliations:** 1 Department of Linguistics, Cognitive Science and Semiotics, Aarhus University, Aarhus, Denmark; 2 Interacting Minds Centre, Aarhus University, Aarhus, Denmark; 3 Center of Functionally Integrative Neuroscience, Aarhus University Hospital, Aarhus, Denmark; Leiden University, NETHERLANDS

## Abstract

Demonstrative reference is central to human communication. But what influences our choice of demonstrative forms such as “this” and “that” in discourse? Previous literature has mapped the use of such “proximal” and “distal” demonstratives onto spatial properties of referents, such as their distance from the speaker. We investigated whether object semantics, and specifically functional properties of referents, also influence speakers’ choices of either demonstrative form. Over two experiments, we presented English, Danish and Italian speakers with words denoting animate and inanimate objects, differing in size and harmfulness, and asked them to match them with a proximal or a distal demonstrative. Objects that offer more affordances for manipulation (smaller and harmless) elicited significantly more proximal demonstratives. These effects were stronger for inanimate referents, in line with the predictions of sensory-functional views on object semantics. These results suggest that demonstrative use may be partly grounded on manual affordances, and hints at the possibility of using demonstratives as a proxy to investigate the organization of semantic knowledge.

## Introduction

### Spatial demonstratives: Function and deictic nature

Spatial demonstratives are referencing expressions present in all languages [1]. They are among the first items to be acquired in early development [1–6]. Moreover, demonstrative determiners (e.g. *this*, *that* in English) and adverbials (*here*, *there*) are by far the most prominent items in the lexicon of children between 1 and 2 years of age, with *that* being the most frequent words for this age group in the CHILDES database [7]. Alongside their role in language development, demonstratives are also among the most frequent words in adults’ lexicon [8]. While not explicitly encoding any specific information about the referent, words like *this* and *that* are used to establish a joint focus of attention on concrete objects in the physical space (*exophoric* usage), or on items in discourse space (*endophoric* usage) [1, 9–12]. The ability to coordinate attention on a referent is a central building block of human social interaction and enables mutual engagement in shared practices [13]. This pivotal function in communication grants demonstratives a special status among the variety of referencing tools languages are endowed with.

Interestingly, both production and comprehension of spatial demonstratives hinge upon the perceptual context of the utterance and the multimodal communicative signals they co-occur with. When used to denote entities in the physical environment, demonstratives are usually coupled with a pointing gesture which enables disambiguation of the intended referents among competing objects [14–17]. Given their tight link with visuo-motor communicative signals and their role in supporting fundamental cognitive processes, such as managing joint attention, it has been hypothesized that demonstratives might be primordial elements in the emergence of language [7]. Indeed, differently from all other function words, the deictic roots of demonstratives cannot be traced back to any content words, which provides evidence in favor of demonstratives having emerged very early in language evolution [7].

Crucially, spatial demonstratives are deictic expressions, i.e. lexical items that differ from other referencing expressions (such as nouns) in that they do not unambiguously denote the intended object, but rather provide *instructions* on how to locate it among various competing referents. Without any contextual information, the meaning of words such as *this* or *that* is opaque: knowledge of some elements of the context of utterance is therefore required in order to single out what they are meant to refer to. In the case of spatial demonstratives, such knowledge can either consist of information on the perceptual context of the utterance (e.g. position of speaker, addressee and/or other objects), or on discourse context (e.g. common ground between interlocutors) [18].

Demonstratives do not carry univocal information on the intended referent, but demonstrative systems across all languages have multiple lexical forms, which encode some deictic contrast facilitating reference resolution [1]. All languages encode at least one distinction between so-called proximal and distal demonstratives, either by explicit lexical forms, such as in the contrast between *this* and *that* in English, or via reinforcing elements, such as in the contrast between the Danish expressions “den *her”* and “den *der”* [1]. Some languages have more complex systems with additional deictic distinctions. In the simplest, dyadic case, the use of either a proximal or a distal form is likely to convey information about the distance of the position of the referent within the search space (e.g. close or far from the speaker). Complex systems including three or more distinct demonstrative forms either lexicalize more fine-grained information on the distance of the referent relative to the speaker (e.g. medial distances), or the location of the referent relative to the addressee, or may even explicitly encode perceptual features of the referent such as its visibility [4].

### This or that: Distance-based or functional contrast?

It is thus widely accepted that the distance between the referent and the speaker and/or the addressee is a crucial factor when it comes to describing the usage criteria of proximal and distal demonstrative forms (but see [19–21] for complementary perspectives). However, the detailed usage profile of the proximal/distal contrast is still a subject of debate, receiving more and more attention in experimental literature.

In a series of experiments, Coventry and colleagues [22–23] have uncovered a mapping between the proximal/distal contrast and the functional organization of space into peripersonal and extrapersonal space, that is, into space within and outside manual reach. Results are consistent across several studies targeting a number of genetically heterogeneous languages. Interestingly, the mapping between referent location and demonstratives seems to be sensitive to the same dynamic adjustments that can modify the boundaries between extrapersonal and peripersonal space. The use of tools, ownership of the referent, and familiarity seem to affect the scope of the proximal deictic space [22–24].

In a reaching task involving hand movements, Bonfiglioli and colleagues [25] have further explored the relationship between functional organization of space and demonstrative reference using semantic priming. In the study, participants were primed with either a proximal or a distal demonstrative, and then asked to reach for objects placed at two possible distances within the participant’s reach. By looking at reaction times in the initiation of reaching movements, they observed semantic interference effects when the demonstrative used for priming and the object’s location were incongruent. This suggests that the contrastive nature of demonstrative systems is sensitive to relative distances between competing referents even within peripersonal space. Additionally, in a recent study [26], we have shown that, when presented with competing referents in a two-dimensional plane extending away from the participant, he/she displays a lateralized bias for proximal demonstratives in favor of the pointing hand. This asymmetry in the organization of space in spatial deixis suggests that the frame of reference for distance-based demonstrative contrasts might be centered on the dominant hand, rather than on the head or the locus of foveal fixation.

Taken together, this corpus of experimental evidence converges on showing that the use of demonstratives could be grounded in representations of objects in terms of their functional properties, including the extent to which they allow manual interaction. In this respect, distance from the speaker is only one of the relevant factors. As we will explore in the next section, a range of semantic features can shape the functional profile of a referent, and therefore possibly modulate lexical preferences for different demonstrative forms.

### Spoons, rockets and other dangerous things: Object semantics and demonstrative use

In spite of a growing interest in the variety of factors that can modulate preferences for proximal or distal demonstratives (e.g. object *token* properties such as familiarity, visibility and ownership [23]), previous studies have largely considered semantic features of the referent (i.e. related to object *type*) as irrelevant to the usage profile of spatial demonstratives.

As outlined above, however, there is increasing evidence suggesting that the distinction between proximal and distal demonstratives maps onto differences in the extent to which referents afford grasp and/or manipulation. Arguably, such affordances do not only depend on whether an object is located within reach, but also on its physical properties, such as object size, and on more abstract properties, such as its harmfulness, or its familiarity.

Therefore, if the assumption that demonstrative use is sensitive to gradient manual affordances holds true, it can be expected that a preference for proximal demonstratives would be observed for referents whose semantic features result in greater affordances for manipulation, regardless of their position in space.

As mentioned, lower-level properties such as size are intuitively some of the core dimensions in shaping an object’s functional profile. The ability to manipulate an object obviously depends on the object being small enough to be graspable by a human hand. If demonstrative use is indeed tied to manual affordances, then smaller objects should be more likely to be referred to via a proximal demonstrative than bigger objects, as smaller objects tend to afford manipulation more easily than bigger ones.

Alongside lower-level properties, additional cognitive dimensions are also directly relevant to manual affordances. It has been pointed out that the functional organization of space into peripersonal and extrapersonal space responds not only to the need for representing action possibilities, but also to defensive purposes [27]. Indeed, it has been found that object valence can interact with perceptual judgements of reachability [28–29]. Moreover, harmfulness can modulate space and object perception in peripersonal space. Peripersonal space tends to shrink for defensive purposes when harmful or undesirable objects are present, and it has been argued that common neural resources might be responsible for modulating spatial attention in peripersonal space, monitoring harmful events, and selecting and coordinating defensive behavior [30–34]. Furthermore, reachability judgements tend to be influenced by the on-line relationship between the object and the subject. The extension of peripersonal space is reduced more substantially when harmful objects are oriented towards the subject compared to when oriented away, regardless of the perceived degree of harmfulness [35]. As demonstrative use is grounded in a dynamic functional organization of space oriented to manual grasp [22–24, 26], it is reasonable to expect that the degree of harmfulness of a referent could modulate the usage profile of spatial demonstratives. More specifically, if these hypotheses hold true, stimuli perceived as dangerous should be more likely to trigger the use of a distal demonstrative than harmless referents.

### The animate-inanimate distinction: A sensory-functional prediction

In addition to properties such as size and harmfulness, existing literature suggests that the distinction between animate and inanimate objects could be another relevant window into the relation between semantics and demonstrative contrasts.

The distinction between animals and artefacts has gained considerable attention in the scientific literature, as it seems to be one of the main dimensions along which human semantic knowledge is organized. Since the 1980s, several neuropsychological studies have shown that semantic knowledge of animate and inanimate objects can be selectively impaired as a consequence of focal brain lesions [36–38]. However, the exact profile of such dissociations and the underlying causes are far from uncontroversial.

In a *domain-specific* interpretation, the dissociation in cognitive deficits reported in the literature is claimed to reflect an evolved modular organization of semantic knowledge with domain-specific systems, supported by separate neurobiological mechanisms [39].

On the other hand, sensory-functional theories (SFT) hypothesize that the observed dissociations in semantic knowledge for living and non-living things can be explained in terms of lower-level sensory and functional properties of objects [38, 40–43]. Sensory-functional theories explain the observed dissociations in terms of the relative importance of sensory and functional properties in the representation of living and non-living things. While sensory features are relatively more important in discriminating between living things, functional properties of objects are attributed more importance in discriminating between different types of artefacts. The observed animate/inanimate dissociation in semantic knowledge is thus explained by a distinction between sensory processing (damage to which primarily results in impaired knowledge for animate beings) and functional representations, where lesions disproportionally impair semantic knowledge for tools and artefacts [38]. More recent formulations of the sensory-functional approach to semantic knowledge have reframed the distinction between animate and inanimate beings in terms of differences in the ratio between sensory and functional features relevant to the representation of category tokens [44]. This ratio is claimed to be larger for animate beings compared to artefacts, as functional features tend to be more prominent in the representation of and discrimination between inanimate objects, while sensory features are equally relevant for the representation of both categories.

Sensory-functional approaches to semantic knowledge have interesting implications with regards to the relationship between object semantics and spatial demonstratives. First, the distinction between animate and inanimate things can itself be thought of as a distinction between objects affording manual interaction to a smaller or greater extent respectively. Intuitively, inanimate objects are often more readily represented in terms of their possibility for manipulation, compared to animate beings. A reasonable hypothesis thus is that a higher proportion of proximal demonstrative would be observed for inanimate referents, compared to animate referents. However, sensory-functional theories additionally posit that representations of inanimate objects are more sensitive to variability along functional dimensions of the feature space than representations of animate objects. If this hypothesis holds true, then differences in functional features, such as size and harmfulness, should determine larger differences in the proportion of proximal *vs* distal demonstratives between tokens of the inanimate category, compared to those observed between animate beings. In more concrete terms, the possibility for interaction with animals is less determined by their size and harmfulness than inanimate objects (small, harmless animals also tend to run away…). Crucially, testing such predictions in an experimental fashion would not only contribute to elucidating the usage profile of spatial demonstratives, but also provide insights on core questions of the debate on the organization of human semantic knowledge.

### The present study

In the present study, we employed a simple elicitation paradigm in order to investigate whether semantic properties of objects, i.e. differences in affordance for manual interaction, systematically influence speakers’ preferences for proximal or distal demonstrative forms.

We tested speakers of English, Danish and Italian over two experiments. These three languages all have dyadic demonstrative systems, that is, they explicitly encode a simple binary contrast between so-called *proximal* and *distal* demonstrative forms. The choice of a cross-linguistic sample was motivated by the aim of countering language specific phenomena, rather than by expectations for cross-linguistic differences in patterns of demonstrative use.

The experiments were distributed in the form of a multiple-choice online survey. Participants were presented with concrete nouns and asked to pair the words with either a proximal or a distal demonstrative, based on their first and most immediate preference. No further linguistic or perceptual context was provided. This was meant to rule out possible confounds due to contextual or co-textual effects, so that observed systematic preferences could only be driven by properties intrinsic to the stimulus words.

Stimulus words differed along three main semantic dimensions denoting either: 1) animate or inanimate referents; 2) big or small referents; 3) harmful or harmless referents.

Levels of these three semantic dimensions involve a common distinction in the degree to which referents afford manipulation. Small referents are likely to offer more manual affordances than big referents. The same holds for harmless compared to harmful referents, and for inanimate compared to animate referents.

Consequently, we expected a preference for proximal demonstratives: 1) for nouns denoting small referents compared to big referents; 2) for nouns denoting harmless referents, compared to harmful referents; 3) for nouns denoting inanimate referents compared to animate referents. We expected to observe these effects as main effects of each of the variables of interest.

Moreover, in line with the sensory-functional view on semantic knowledge, we predicted that the effect of harmfulness and size would be stronger for inanimate objects, compared to animate beings. We therefore expected to observe two-way interactions between animacy and harmfulness, and between animacy and size. We expected our predictions to hold across the three languages tested in the experiment.

The aim of the study was two-fold. On the one hand, we aimed at contributing to elucidate the usage profile of the proximal/distal demonstrative contrast. On the other hand, we aimed at exploring the possibility of using demonstratives to probe the organization of human semantic knowledge.

As mentioned, the study is articulated into two experiments. The first study tested speakers of all three languages (Experiment 1). We then replicated it in Italian and Danish (Experiment 2) using the same design and procedure, but with a different stimulus set.

Data and code are available on the Open Science Framework at osf.io/gnh5s.

## Experiment 1

### Methods

#### Participants

In Experiment 1, we collected data from 131 English speakers (96 native), 102 Danish speakers (101 native), and 131 Italian speakers (126 native).

Participants were recruited online. The survey was advertised via social media and institutional web platforms. No information on the purpose of the experiment was provided in advance. Participants took part in the experiment voluntarily, and they did not receive monetary compensation for their participation.

At the beginning of the study, participants were provided with instructions and a detailed consent form, and they consented to the conditions of participation by proceeding to the first experimental trial. At the end of the experiment, participants were asked to provide information on their gender and age. Moreover, they were asked to specify whether they were native speaker of the language of the survey. If this was not the case, they were further asked to specify their native language. In the English version of the experiment, those who stated having English as their native language were asked to specify which variety of English they spoke, choosing between American English, British English, or other. Due to the limitations of our convenience sampling method, this information was not included in the analysis.

Participants were further asked to specify whether they knew the meaning of all the words presented in the survey. If not, they were asked to tick the words they did not know, and the corresponding data points were then excluded from the analysis. Only data from participants who completed the survey were included in the analysis.

As two participants from the English dataset reported not to know the meaning of eight out of thirty-two experimental words, we excluded them from the analysis. All other participants reported to know the meaning of the vast majority of words (median: 40 out of 40 words, range: 36–40), thus no further data were discarded. Both L1 and L2 speakers were included in the analysis.

The study received ethical approval from the Ethical Committee of the Cognition and Behaviour Lab at Aarhus University, Denmark.

#### Platform

The experiment was hosted by the online platform *Qualtrics Experience Management Platform* (Qualtrics, Provo, UT, USA). Access to the platform was provided by the School of Business and Social Sciences (BSS) at Aarhus University.

#### Task and procedure

Participants in both experiments were presented with 40 individual words. Participants had to match each word with either a proximal or a distal demonstrative, that is, *this* or *that* in English, *den/det her* or *den/det der* in Danish, and *questo/a* or *quello/a* in Italian. Target words were displayed one at a time at the top of the screen together with the two possible demonstrative forms positioned on two separate lines below the word. Participants chose between the two demonstrative forms by ticking the box corresponding to the preferred demonstrative form. They were asked to make their choice based on their first and immediate preference. No further context nor any extra information was displayed on screen.

After having made their choice, participants could proceed to the next word. It was not possible to go back to previous trials once the response was given. A response was required in order to proceed to the next trial. The order of presentation of nouns was randomized across participants. The order in which the two demonstratives were displayed on screen was randomized across trials and participants.

#### Stimuli

All the words were singular nouns. Out of 40 nouns, 32 were experimental words, and 8 were fillers. All the words used for Experiment 1 are reported in Table 1 (in English). The full stimulus list in all three languages is reported in the Supporting Information (S1 Table).

**Table 1:** Stimulus words for Experiment 1, English.

**Animate**		
	*Big*	*Small*
*Harmful*	Bull, shark, hyena, snake	Bee, flea, spider, rat
*Harmless*	Camel, goose, lamb, penguin	Cricket, kitten, robin, shrimp
**Inanimate**		
	*Big*	*Small*
*Harmful*	Bomb, jail, rocket, rifle	Burner, dagger, needle, thorn
*Harmless*	Bench, couch, cradle, tent	Coin, comb, cookie, soap
**Fillers**		
Deal, dawn, hurry, loss, quarrel, rise, rest, stress		

Experimental words always referred to concrete objects. *Animacy* (animate/inanimate), *Size* (big/small) and *Harmfulness* (harmful/harmless) of the referent were the binary variables of interest, yielding 2x2x2 combinations per language. Participants were presented with four words from each possible combination, which resulted in a within-subject repeated measures design. Fillers denoted abstract entities.

Target words were chosen from a semantic knowledge database (in English) including 1000 concrete nouns rated along 218 semantic dimensions on an integer scale from 1 to 5 [45].

For the purpose of the study, words were selected according to their ratings on the dimensions “Is it an animal?”, corresponding to the variable *Animacy* in our experimental design, “Is it bigger than a loaf of bread?”, corresponding to the variable *Size*, and “Is it dangerous?”, corresponding to the variable *Harmfulness*. Different dimensions were available which could be relevant for the variable *Size* relying on comparisons in size between the target objects and different referent objects. Among them, however, we chose the dimension that best mirrored a distinction between objects whose size allows manual grasp and objects whose size does not.

All nouns labelled as non-animate in our experiment were rated less than 3 along the *Animacy* dimension, while animate referents were rated 3 or more. Nouns labelled as small referents were rated less than 3 on the Size dimension, while big referents were rated 3 or more. Nouns denoting dangerous referents were rated 3 or more on the *Harmfulness* dimension, while harmless referents were rated less than 3. All words were translated into Italian and Danish by native speakers of the two languages (the paper’s first and last authors). Danish translations of a subset of the words present in the database had been previously used and validated in the context of a neuroimaging study on word processing [46].

#### Demonstrative expressions

In English, participants could choose to match the noun with either “this” or “that”.

In Danish, demonstratives in their adjectival use were created by combining the articles “den” or “det” (roughly equivalent to “it” in English) with the demonstrative adverbs “her” (“here”, in English) or “der” (“there”, in English). As Danish has two grammatical genders, demonstratives were matched to the grammatical gender of the noun. For nouns in common gender, participants could choose between “den her” and “den der”, while for nouns in neutral gender, “det her” and “det der” were presented as possible options.

Italian also has two grammatical genders, and demonstratives in their adjectival use have to be matched for the gender of the noun. For nouns starting with a consonant sound, “questo” and “quello” / “quel” are respectively the proximal and distal masculine forms, while “questa” and “quella” are the feminine forms. The masculine demonstrative “quel” is used for all nouns except those starting with semi-consonantic sounds (*i*, *y* and *j*), with sibilants (*s* and *z*), and with other consonant clusters (*gn*, *sc*, *pn* and *ps*), where “quello” is the correct form. For nouns starting with a vowel, the final vowel of both feminine and masculine demonstratives “questo” / “questa” and “quello” / “quella” is elided, yielding the forms “quest’” and “quell’” respectively. For example, when the feminine demonstratives “questa” and “quella” are to be coupled with the feminine noun “anatra” (duck), the resulting form would be “quest’anatra” or “quell’anatra”. For Italian, we decided to uniformly present the forms “questo” / “quello” for masculine words and “quello” / “quella” for feminine words regardless of the first letter of the noun. We decided to do so in order to obtain uniformity of methods across languages, and because we anticipated that, given the presentation format, presenting the elided forms “quest’” and “quell’” not followed by a noun would have appeared strange to a proficient speaker, even though such forms would have been the grammatically correct ones.

#### Word frequency

In order to control for potential confounds due to effects of word frequency, we compared frequency of experimental words across levels of each of the experimental factors. For English, we extracted word frequencies from the British National Corpus [8]. For Italian and Danish, we extracted word frequencies from the respective corpora in the TenTen Corpus Family [47] from 2017. Details on the distribution of word frequencies for Experiment 1 are provided as Supporting Information (S1 Fig).

Linear regressions with animacy, harmfulness and size and their interactions as fixed effects and word frequency as outcome variable revealed no significant differences (p > .05) across levels of the experimental variables in any of the three languages.

#### Analysis

Animacy, harmfulness and size were used as binary independent variables.

For the independent variable *animacy*, nouns were coded as either *inanimate* or *animate*. *Inanimate* was set as reference level. For the independent variable *Size*, nouns were coded as either *small* or *big*. *Small* was set as reference level. For the independent variable *Harmfulness*, nouns were coded as either *harmless* or *harmful*. *Harmless* was set as the reference level. For the independent variable *Language*, Danish was set as reference level.

The outcome variable coded for the demonstrative chosen at each trial. Responses were coded as either *distal* or *proximal*. *Distal* was set as reference level, while *proximal* was coded as success outcome. All instances of “this” in English, “den/det her” in Danish, and “questo” / “questa” in Italian were coded as *proximal* demonstratives, while all instances of “that” in English, of “den/det der” in Danish, and “quello” / “quella” in Italian were re-coded as *distal* demonstratives.

Data visualization and analysis was performed using RStudio, version 1.1.383 (RStudio Team 2016).

Data were analyzed using mixed-effects logistic regression implemented via the function *glmer* from the package *lme4* [48]. Parameters for the logistic regression model were estimated using maximum likelihood estimation with Laplace approximation, and inferences were drawn via likelihood ratio tests. R^2^ estimates reported in the analysis and discussion are computed using the function r2 from the R package *sjstats* [49].

Data from all languages were analyzed in a single logistic regression model. The fixed effects structure included all the three variables of interest, a categorical predictor coding for language, and the full interaction structure. The random effects structure included a random intercept for participants.

### Results

**Fig 1:**
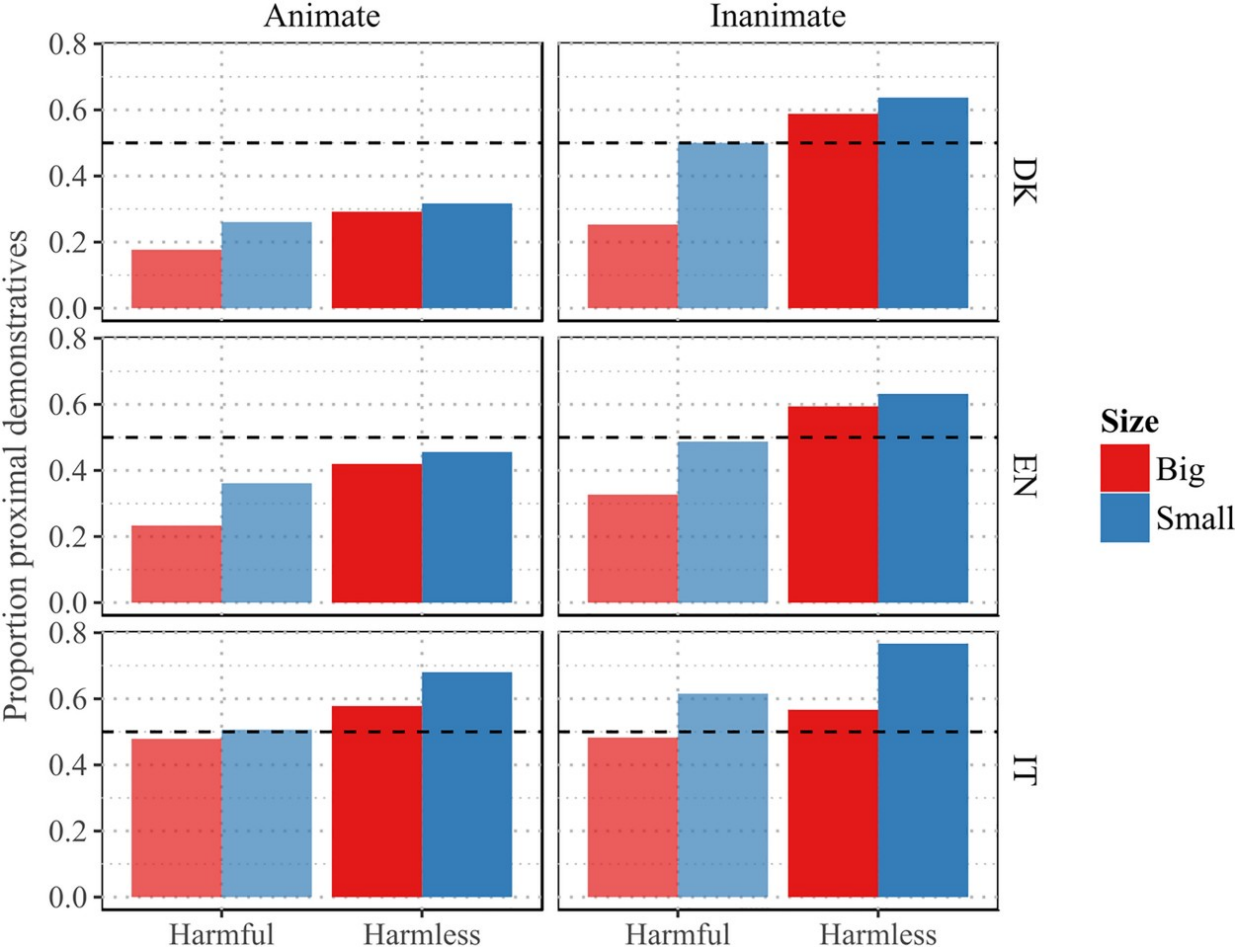
Proportion of proximal demonstratives across languages, Experiment 1. Small, inanimate and harmless nouns tend to be denoted with a proximal demonstrative across languages. The effects of size and harmfulness tend to be stronger for inanimate, compared to animate referents.

Fig 1 provides an overview of the data from Experiment 1.

The analysis revealed an overall preference for distal demonstratives, in line with corpus frequency data for each of the three languages, β = -1.75, se = 0.16, z = -10.98, p < .001.

There was a significant main effect of language, indicating that the proportion of distal demonstratives was overall larger in Danish compared to English, β = 0.43, se = 0.20, z = 2.13, p < .05, and compared to Italian, β = 1.65, se = 0.2, z = 8.41, p < .001. The model revealed a significant main effect of harmfulness, β = 0.74, se = 0.18, z = 4.1, p < .001, and a significant main effect of size, β = 0.56, se = 0.18, z = 3.07, p < .01, both in the predicted direction. Additionally, there was a main effect of animacy, β = 0.52, se = 0.18, z = 2.82, p < .01, as well as significant interactions between animacy and harmfulness, β = 0.91, se = 0.24, z = 3.76, p < .001, and animacy and size, β = 0.68, se = 0.24, z = 2.79, p < .01, suggesting that the effects of harmfulness and size were more pronounced for inanimate objects than for animate beings.

The analysis also hinted at unpredicted cross-linguistic differences in the effects of the experimental variables. There was a significant interaction between language and animacy, showing that the effect of animacy was stronger in Italian compared to Danish, β = -0.49, se = 0.22, z = -2.21, p < .05. Moreover, the interaction between animacy and harmfulness was significantly stronger in Danish, compared to English, β = -0.64, se = 0.31, z = -2.07, p < .05, and Italian, β = -0.98, se = 0.31, z = -3.21, p < .01, as shown by the three-way interaction between animacy, harmfulness and language. A three-way interaction between animacy, size and language indicated that the interaction between animacy and size was marginally less pronounced in English, compared to Danish, β = -0.62, se = 0.31, z = -1.97, p < .05.

The model also displayed an unpredicted three-way interaction between animacy, size, and harmfulness, β = -0.81, se = 0.33, z = -2.46, p < .05, as well as a four-way interaction between animacy, size, harmfulness and language, when comparing Danish to Italian, β = 1.09, se = 0.43, z = 2.56, p < .05. The model has a marginal R^2^ of 0.126, and a conditional R^2^ of 0.265.

A summary of the estimated fixed effects coefficients is reported in Table 2.

**Table 2:** Fixed effects for Experiment 1.

	Beta	SE	z	95% CI lower	95% CI upper	Odds Ratio	p
(Intercept)	-1,75	0,16	-10,98	-2,06	-1,44	0,17	< .001***
Animate	0,52	0,18	2,82	0,17	0,87	1,68	< .01 **
Size	0,56	0,18	3,07	0,21	0,91	1,75	< .01 **
Harm	0,74	0,18	4,1	0,39	1,09	2,1	< .001***
Lang (EN vs. DK)	0,43	0,2	2,13	0,04	0,82	1,54	< .05 *
Lang (IT vs. DK)	1,66	0,2	8,42	1,27	2,05	5,26	< .001***
Animate x Size	0,68	0,24	2,79	0,21	1,15	1,97	< .01 **
Animate x Harm	0,91	0,24	3,76	0,44	1,38	2,48	< .001***
Size x Harm	-0,11	0,24	-0,44	-0,58	0,36	0,9	n.s.
Animate x Lang (EN vs. DK)	-0.002	0,23	-0,01	-0,45	0,45	1	n.s.
Animate x Lang (IT vs. DK)	-0,5	0,23	-2,21	-0,95	-0,05	0,61	< .05 *
Size x Lang (EN vs. DK)	0,12	0,23	0,53	-0,33	0,57	1,13	n.s.
Size x Lang (IT vs. DK)	-0,26	0,22	-1,14	-0,69	0,17	0,77	n.s.
Harm x Lang (EN vs. DK)	0,22	0,23	0,95	-0,23	0,67	1,25	n.s.
Harm x Lang (IT vs. DK)	-0,28	0,22	-1,28	-0,71	0,15	0,76	n.s.
Animate x Size x Harm	-0,81	0,33	-2,46	-1,46	-0,16	0,44	< .05 *
Animate x Size x Lang (EN vs. DK)	-0,62	0,31	-1,97	-1,23	-0,01	0,54	< .05 *
Animate x Size x Lang (IT vs. DK)	-0,43	0,31	-1,41	-1,04	0,18	0,65	n.s.
Animate x Harm x Lang (EN vs. DK)	-0,64	0,31	-2,07	-1,25	-0,03	0,53	< .05 *
Animate x Harm x Lang (IT vs. DK)	-0,98	0,31	-3,21	-1,59	-0,37	0,38	< .01 **
Size x Harm x Lang (EN vs. DK)	-0,41	0,31	-1,33	-1,02	0,2	0,66	n.s.
Size x Harm x Lang (IT vs. DK)	0,3	0,31	0,97	-0,31	0,91	1,35	n.s.
Animate x Size x Harm x Lang (EN vs. DK)	0,76	0,43	1,78	-0,08	1,6	2,14	n.s.
Animate x Size x Harm x Lang (IT vs. DK)	1,1	0,43	2,57	0,26	1,94	2.99	< .05 *

### Interim discussion

Data from the three languages all converge on showing a strong, significant main effect of harmfulness and size in the direction predicted in the introduction. The proportion of proximal demonstratives over distal demonstratives was consistently higher in the case of harmless referents compared to harmful referents, and in the case of smaller referents compared to big referents. This is in line with our hypothesis that the proximal/distal demonstrative contrast encodes a gradient distinction in the extent to which objects lend themselves to manual interaction.

A main effect of animacy was also detected, but the strength of such effect seemed to vary across languages. Additionally, the model provided strong evidence in favor of the predicted interaction between animacy and size and between animacy and harmfulness, though such interactions were significantly more robust in Danish compared to English and Italian. Taken together, the results lend support to all our predictions suggesting several, motivated semantic dimensions influence speakers’ choice of demonstratives.

The results from Experiment 1, however, leave a number of questions open to discussion.

First, we did not expect to observe strong cross-linguistic differences in the effects of interest. Based on these findings, it cannot be excluded that cross-linguistic discrepancies are due to actual differences in patterns of demonstrative use across the three languages of interest, but a number of alternative explanations are compatible with the observed patterns. Bigger sample sizes, which increase statistical power, might provide more robust insights on cross-linguistic differences in patterns of demonstrative use.

Secondly, as explained in section 2.1.5, the matching between the default distal demonstrative forms presented in the survey and stimulus words was sometimes not entirely correct from a grammatical point of view in the Italian version of the experiment. A separate analysis of the Italian data, reported in S1 Appendix, showed that this affected participants’ choices for proximal or distal demonstrative forms, with a significantly higher proportion of proximal demonstratives for cases in which the matching between stimulus word and distal demonstratives was experienced as strange. It can therefore not be ruled out that the differences between Danish and Italian data detected in the analysis could potentially be driven by such confound.

Furthermore, the unexpected three-way interaction between animacy, size and harmfulness is difficult to relate to predictions from the present study, or to expectations drawn from the literature. While it could be explained in terms of the psychological prominence of the harmfulness dimension, with the interaction between size and animacy being amplified in the case of harmful referents, such an effect still remains difficult to interpret and highlights the potential for false positives due to our complex model. Given the relatively low number of stimulus words, it also could not be excluded that such effect could be driven by particular words, more than by the manipulations in semantic features.

In order to test the reliability and replicability of the effects detected in experiment 1, we decided to collect data from a second set of stimulus words. In the second experiment, we aimed at bigger sample sizes in order to increase the statistical power of the analysis. The availability of a second dataset also enabled us to conduct a cumulative analysis of the two datasets. The availability of more stimulus words for the cumulative analysis provided the possibility of fitting a more complex and more conservative random effects structure, including intercepts for each stimulus word, in order to rule out the possibility of the observed effects being driven by specific stimulus words.

## Experiment 2

### Methods

#### Participants, task, design and procedure

In Experiment 2, we collected data from highly proficient Italian speakers and Danish speakers. In this experiment, 291 Italian speakers (280 native) and 225 Danish speakers (219 native) completed the survey. Recruitment and data collection followed the same procedure and conformed to the same criteria as Experiment 1.

The task, procedure and modality of stimulus presentation were analogous to Experiment 1. However, given the potential confounds present in the Italian dataset from Experiment 1, in Experiment 2 we only presented words which would not require vowel elision in Italian. Moreover, in the Italian version of the survey we presented “quel” as option for distal demonstratives when required, this time prioritizing grammatical correctness over uniformity of stimulus presentation.

#### Stimulus set

For Experiment 2, we initially selected 32 experimental words on a qualitative basis. The design was identical to experiment 1, with 2x2x2 combinations of levels of the three experimental factors per language. We selected four words per each combination in the design matrix. The English translations of all the nouns used in Experiment 2 are reported in Table 3. The corresponding Danish and Italian words are reported as Supporting Information (S2 Table).

**Table 3:** Stimulus words for Experiment 2 (English translation).

**Animate**		
	*Big*	*Small*
*Harmful*	Tiger, cobra, crocodile, boar	Mosquito, jellyfish, tarantula, tick
*Harmless*	Giraffe, horse, dolphin, fawn	Rabbit, butterfly, squirrel, sparrow
**Inanimate**		
	*Big*	*Small*
*Harmful*	Meteorite, avalanche, volcano, bomber	Mine, poison, bullet, knife
*Harmless*	School, oak, double-bass, dome	Mug, shoe, spoon, apple
**Fillers**		
Change, defeat, disappearance, disappointment, peace, promotion, shame, surprise, shame		

As in Experiment 1, none of the experimental words were consistently reported as unknown to participants (median = 40 words known across participants, range: 37–40), and all participants were included in the analysis.

In order to obtain quantitative scores on each of the three dimensions of interest, comparable to the scores for stimuli from Experiment 1 in Sudre and colleagues’ [45] database, and in order to ensure that qualitative word selection was not severely biased, we derived semantic ratings for each of the words from word embeddings via Support Vector Regression.

Using the *svm()* function from the *e1071* library in RStudio [50], we trained three SVR models on the semantic dataset from Sudre and colleagues [45]. We extracted 300-dimensional GloVe vector representations [51] trained on Wikipedia and Gigaword for all words in the dataset (available for download here: https://nlp.stanford.edu/projects/glove/). All the 300 dimensions of the vectors were used as features to tune and train the SVR model. Scores for animacy, harmfulness and size were the variables to be predicted. We tuned model parameters for values of epsilon ranging from 0 to 1 (with 0.1 steps) and cost values ranging from 2^2^ to 2^9^. The best performing model for each of the variables of interest was used to predict animacy, size and harmfulness scores for experimental words, once again using 300-dimensional vector representations as features. We performed a median split on the predicted ratings for each of the categories of interest, and we assessed the performance of the model in terms of the number of words ending up in the correct half of the value range.

To provide a concrete example, the models predicted scores for animacy on a scale from 1 to 5. All animate objects were expected to display values higher than the median. If any animate object displayed a value lower than the median, or any inanimate objects displayed a value higher than the median, this was subtracted from the models’ performance. The model for animacy achieved 100% accuracy. The model for harmfulness achieved 81.25% accuracy. The model for size achieved 87.5% accuracy. Such performance scores can be considered as sufficient evidence for word selection being unbiased and reliably consistent with the criteria and metrics from experiment 1.

The predicted scores were later used for the parametric analysis of the data reported in S2 Appendix.

**Fig 2:**
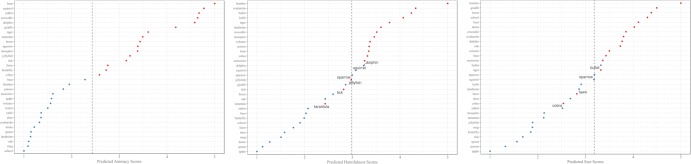
**Scores for Animacy (2a), Harmfulness (2b) and Size (2c), predicted by SVR.** Labelled items are words that do not conform to our qualitative binary classification.

The ratings predicted by the SVR models are plotted in Fig 2

#### Word frequency

Details on the distribution of word frequencies for Experiment 2 are displayed in S2 Fig. As for Experiment 1, linear regressions for word frequencies did not display any significant differences between categories in any of the three languages (p>.05).

#### Analysis

We analyzed the data in a logistic mixed-effects regression model. As for Experiment 1, the fixed effects structure included binary predictors for Animacy, Harmfulness and Size, a categorical predictor coding for Language, and the full interaction structure. The random effects structure included random intercepts for each participant. Danish was set as reference level for the categorical regressor coding for language.

### Results

**Fig 3:**
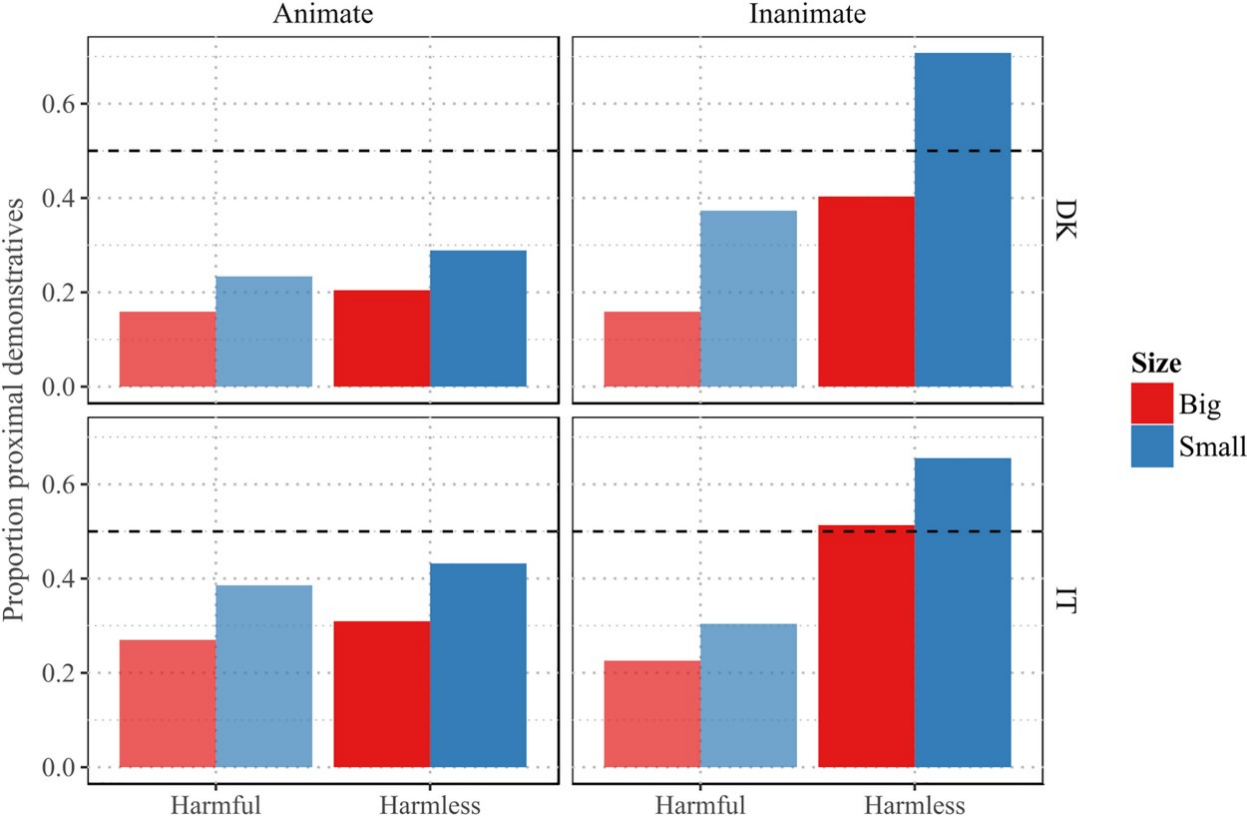
Overview of the data from Experiment 2. Small and harmless objects display a tendency to be denoted with a proximal demonstrative across languages. The effects of size, and harmfulness interact with animacy.

An overview of the data from Experiment 2 is provided in Fig 3.

The analysis displays an overall preference for distal demonstratives, β = -1.91, se = 0.1, z = - 18.24, p < .001. There was a significant main effect of language, with a higher proportion of proximal demonstratives for Italian compared to Danish, β = 0.79, se = 0.13, z = 5.97, p < .001.

As in Experiment 1, there was a significant main effect of Harmfulness, β = 0.31, se = 0.12, z = 2.55, p < .05 in the predicted direction, as well as a main effect of Size, β = 0.52, se = 0.12, z = 4.27, p < .001. However, no main effect of Animacy was observed (β = -0.04, se = 0.13, z = -0.31, p > .05).

There was a significant interaction between Animacy and Harmfulness, β = 1.17, se = 0.17, z = 6.77, p < .001, as well as a significant interaction between Animacy and Size, β = 0.6, se = 0.17, z = 3.56, p < .001, suggesting that the difference in proportion of proximal demonstratives between harmful and harmless referents was bigger for inanimate referents, compared to animate referents.

The interaction between Animacy, Size and Language was significant, β = -0.73, se = 0.21, z = -3.43, p < .001, indicating that there was a bigger difference in the effect of size across animate and inanimate beings in Danish, than in Italian. The model achieves a marginal R^2^ of 0.138, and a conditional R^2^ of 0.299.

A summary of the estimated fixed effects coefficients is reported in Table 4.

**Table 4:** Fixed effects from statistical model for Experiment 2.

	Beta	SE	z	95% CI lower	95% CI upper	Odds Ratio	p
(Intercept)	-1,91	0,1	-18,24	-2,11	-1,71	0,15	< .001***
Animate	-0,04	0,13	-0,31	-0,29	0,21	0,96	n.s.
Size	0,52	0,12	4,27	0,28	0,76	1,68	< .001***
Harm	0,31	0,12	2,55	0,07	0,55	1,36	< .05 *
Lang	0,79	0,13	5,97	0,54	1,04	2,2	< .001***
Animate x Size	0,6	0,17	3,56	0,27	0,93	1,82	< .001***
Animate x Harm	1,17	0,17	6,77	0,84	1,5	3,22	< .001***
Size x Harm	0,02	0,17	0,1	-0,31	0,35	1,02	n.s.
Animate x Lang	-0,24	0,16	-1,5	-0,55	0,07	0,79	n.s.
Size x Lang	0,09	0,15	0,57	-0,2	0,38	1,09	n.s.
Harm x Lang	-0,09	0,15	-0,57	-0,38	0,2	0,91	n.s.
Animate x Size x Harm	0,38	0,23	1,64	-0,07	0,83	1,46	n.s.
Animate x Size x Lang	-0,73	0,21	-3,43	-1,14	-0,32	0,48	< .001***
Animate x Harm x Lang	0,06	0,22	0,27	-0,37	0,49	1,06	n.s.
Size x Harm x Lang	-0,03	0,21	-0,14	-0,44	0,38	0,97	n.s.
Animate x Size x Harm x Lang	-0,18	0,3	-0,62	-0,77	0,41	0,84	n.s.

### Interim discussion

The analysis of data from Experiment 2 confirmed the robustness of the main effects of harmfulness and size detected in Experiment 1, and it provided additional strong evidence in favor of the animate-inanimate distinction modulating the effects of size and harmfulness. This, again, is in line with our initial predictions. However, there was no significant main effect of animacy, contrary to what observed in the analysis of data from Experiment 1. The predicted effects of harmfulness and size and the interaction between these two factors and animacy are consistent across both languages, but some of the effects vary in strength across languages. This is also in line with the findings from Experiment 1.

### Overall analysis

In order to further test the robustness of our results, and to provide a comprehensive picture of the results from the two studies, we conducted a cumulative analysis of both data from Experiment 1 and Experiment 2 in a mixed-effects logistic regression model. In this analysis, we fitted the same fixed effects structure used for the individual analyses of Experiment 1 and Experiment 2, but we refined the random effects structure by including intercepts for each individual stimulus word.

The fixed effects structure included categorical regressors for Animacy, Harmfulness, Size and Language, as well as the full interaction structure. The random effects structure included not only one intercept for each participant, but also random intercepts for each individual word, nested within levels of each of the experimental variables.

These latter random effects ensured that the parameter estimates were pooled for specific effects of individual experimental words, thus ruling out the possibility that observed main effects or interactions would be driven only by specific items in the stimulus set, rather than being genuine effects of the experimental manipulation. Given the anomalies detected in the Italian dataset from Experiment 1, this dataset was excluded from the analysis.

Overall, the model included data from 327 Danish speakers (320 native), 291 Italian speakers (280 native), and 131 English speakers (96 native).

As in Experiment 1, Danish was set as reference level for the categorical regressor coding for the language of each experiment. The model revealed an overall preference for distal demonstratives, β = -1.95, se = 0.17, z = -11.31, p < .001. There was a significant main effect of language showing that such preference was stronger in Danish than in Italian, β = 0.83, se = 0.13, z = 6.39, p < .001, and in English, β = 0.59, se = 0.19, z = 3.06, p < .01.

There was a main effect of Harmfulness, β = 0.58, se = 0.23, z = 2.5, p < .05, and a main effect of Size, β = 0.59, se = 0.23, z = 2.58, p < .01. No main effect of animacy was observed (β = 0.27, se = 0.23, z = 1.18, p > .05). The interaction between animacy and harmfulness was significant, β = 1.02, se = 0.32, z = 3.16, p < .01, as well as the interaction between animacy and size, β = 0.65, se = 0.32, z = 2.04, p < .05.

The model revealed a couple of significant differences across languages. There was an interaction between animacy and language, with a smaller difference in distal/proximal demonstratives used for animate *versus* inanimate referents in Italian, compared to Danish, β = -0.37, se = 0.16, z = -2.34, p < .05. The interaction between animacy and size was significantly stronger in Danish, compared to English, β = -0.64, se = 0.30, z = -2.13, p < .05, and Italian, β = -0.76, se = 0.21, z = -3.59, p < .001. The interaction between animacy and harmfulness was significantly stronger in Danish compared to English, β = -0.71, se = 0.3, z = -2.35, p < .05. No other effects reached statistical significance. The overall model with refined random effects structure yields a marginal R^2^ of 0.123 and a conditional R^2^ of 0.319.

A summary of the estimated fixed effects coefficients is reported in Table 5.

**Table 5:** Fixed effects from statistical model for overall analysis.

	Beta	SE	z	95% CI lower	95% CI upper	Odds Ratio	p
(Intercept)	-1,96	0,17	-11,32	-2,29	-1,63	0,14	< .001***
Animate	0,27	0,23	1,18	-0,18	0,72	1,31	n.s.
Size	0,59	0,23	2,58	0,14	1,04	1,8	< .01 **
Harm	0,58	0,23	2,5	0,13	1,03	1,79	< .05 *
Lang (EN vs. DK)	0,59	0,19	3,06	0,22	0,96	1,8	< .01 **
Lang (IT vs. DK)	0,83	0,13	6,39	0,58	1,08	2,29	< .001***
Animate x Size	0,65	0,32	2,04	0,02	1,28	1,92	< .05 *
Animate x Harm	1,02	0,32	3,16	0,39	1,65	2,77	< .01 **
Size x Harm	-0,11	0,32	-0,33	-0,74	0,52	0,9	n.s.
Animate x Lang (EN vs. DK)	0,07	0,23	0,3	-0,38	0,52	1,07	n.s.
Animate x Lang (IT vs. DK)	-0,37	0,16	-2,34	-0,68	-0,06	0,69	< .05 *
Size x Lang (EN vs. DK)	0,12	0,22	0,54	-0,31	0,55	1,13	n.s.
Size x Lang (IT vs. DK)	-0,01	0,15	-0,04	-0,3	0,28	0,99	n.s.
Harm x Lang (EN vs. DK)	0,27	0,22	1,23	-0,16	0,7	1,31	n.s.
Harm x Lang (IT vs. DK)	-0,22	0,15	-1,44	-0,51	0,07	0,8	n.s.
Animate x Size x Harm	-0,17	0,45	-0,37	-1,05	0,71	0,84	n.s.
Animate x Size x Lang (EN vs. DK)	-0,64	0,3	-2,13	-1,23	-0,05	0,53	< .05 *
Animate x Size x Lang (IT vs. DK)	-0,76	0,21	-3,61	-1,17	-0,35	0,47	< .001***
Animate x Harm x Lang (EN vs. DK)	-0,71	0,3	-2,35	-1,3	-0,12	0,49	< .05 *
Animate x Harm x Lang (IT vs. DK)	0,2	0,21	0,92	-0,21	0,61	1,22	n.s.
Size x Harm x Lang (EN vs. DK)	-0,42	0,3	-1,4	-1,01	0,17	0,66	n.s.
Size x Harm x Lang (IT vs. DK)	0,08	0,21	0,39	-0,33	0,49	1,08	n.s.
Animate x Size x Harm x Lang (EN vs. DK)	0,65	0,41	1,56	-0,15	1,45	1,92	n.s.
Animate x Size x Harm x Lang (IT vs. DK)	-0,13	0,29	-0,46	-0,7	0,44	0,88	n.s.

## Post-hoc analyses

### Harmfulness and size as parametric predictors

As a further control test, we analyzed the data using the scores on the harmfulness and size dimensions for each stimulus word as parametric predictors, and demonstrative as outcome variable. Animacy was included as categorical predictor, assuming a binary distinction between animals and inanimate beings. This step was meant to test claims on gradient sensitivity of demonstratives to the experimental variables, and to rule out the possibility that observed effects could be artefacts of the binary categorization of harmfulness and size.

The results of the parametric analysis confirmed all the effects observed in the binary analysis, and they are reported in S2 Appendix.

### Manipulability and experimental variables

The analyses reported in the previous sections elucidate how size, harmfulness and animacy influence demonstrative use. In our experiments, these three dimensions are claimed to be proxies of manipulability, under the hypothesis that manual affordances drive the choice of specific demonstrative forms.

However, while our analyses decompose the effect of manipulability into finer-grained semantic components, the relationship between manipulability and the combination of our experimental variables is more complex than that of a complete overlap.

On the one hand, the three dimensions tested here do not entirely exhaust the range of semantic features contributing to shape an object’s degree of manipulability. To provide a concrete example, shape also plays a role in determining the extent to which an object afford manipulation, a factor which is not accounted for by the combination of our experimental variables. Our models might therefore leave unexplained part of the variance in the data which can still be accounted for by manual affordances.

On the other hand, the three semantic dimensions tested in our experiments are likely to encode *more* semantic content than mere manipulability. This residual information might also have a consistent impact on demonstrative choices. For instance, the distinction between a harmless object and a harmful one does not only translate into a difference in how easy, or how desirable, it is to manually interact with the objects. A harmful and a harmless object also differ in how willing one might be to be exposed to them, or to establish physical proximity. Our analyses do not allow to fully assess to what extent the observed effects are driven by distinctions in manual affordances common to all three experimental variables, rather than by variable-specific (non-shared) semantic content encoded by each feature.

In order to further test our claim that observed effects are driven by manual affordances, we acquired explicit manipulability ratings *post hoc* for each of the words used in Experiment 2. These ratings were used for a two-fold purpose. First, we ensured that variation along the three variables tested in our experiments does indeed reflect significant and consistent distinctions in the extent to which objects afford manipulation. Secondly, we added explicit manipulability scores as a parametric regressor to the model used for the analysis of the Danish data from Experiment 2. This allowed to disentangle the shared effect of manipulability from any potential effects of residual semantic information encoded by each individual semantic variable.

### Manipulability ratings

We asked native speakers of Danish to rate each of the 32 experimental words from Experiment 2 for manipulability. For each word, participants provided three separate scores by answering the following questions: 1) Can you move it with your hands? (“Kan du flytte den med hænderne?"); 2) Is it something you would consider moving with your hands? (“Er det en ting, som du kunne finde på at flytte med hænderne?”); 3) Is it something that would let you move it with your hands? (”Er det noget, der ville lade sig flytte med hænderne”?). These three formulations present the rather abstract problem of how “manipulable” an object is from concrete perspectives, thus confronting participants with specific, explicit and easy-to-grasp problems. The three scores for each word were summed, in order to obtain a reliable and unbiased quantitative proxy to the overarching semantic property of interest.

**Fig 4:**
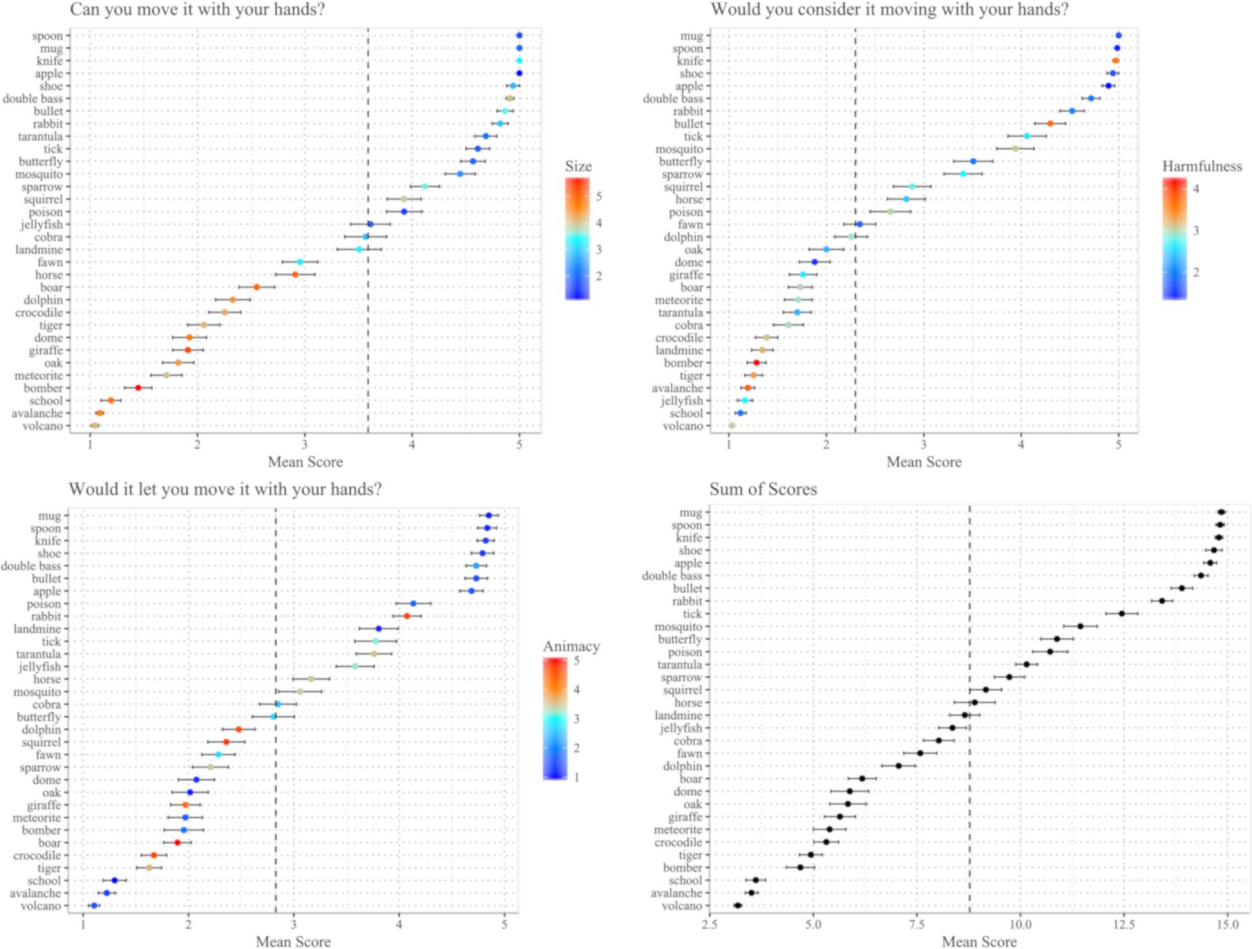
Overview of manipulability scores per word. The figures display mean scores for each word on each of the three questions. Error bars reflect the standard error of the mean. Values in the bottom right panel correspond to the mean (across participants) of the sum of the three scores. Colors indicate the SVR-derived scores along the animacy, size, and harmfulness dimensions used in the parametric analysis.

The questionnaire was set up in Qualtrics, and advertised via social media and institutional web platforms. Before the beginning of the questionnaire, participants were presented with a consent form analogous to the one used for Experiment 1 and 2, and consented to its conditions by proceeding to the first trial. Participants were presented with words in randomized order, one word at a time, and asked to provide a rating on a Likert scale from 1 to 5 in response to each of the three questions. They scored each word by moving the cursor of a slider to an integer value between 1 and 5. We collected data from 67 participants. Fig 4 displays mean scores for each experimental word across the three questions, as well as the sum of all three scores for each word.

### Experimental variables reflect manipulability ratings

In order to test whether different levels of size, animacy and harmfulness reliably reflect distinctions in the degree of manipulability of the referent, we fitted a mixed effects linear regression using binary predictors for size, harmfulness, and animacy, and their interactions, as fixed effects, and the sum of manipulability scores as outcome variable. The model included a random intercept for each participant.

The model displays a significant main effects of harmfulness, β = 1.18, se = 0.27, t(2068.95) = 4.37, p < .001, suggesting that objects coded as “harmful” score significantly lower on manipulability compared to harmless objects. The model also displays a significant main effect of size, β = 4.49, se = 0.27, t(2068.95) = 16.64, p < .001, suggesting that objects coded as “big” are rated as less manipulable than objects coded as “small”. Interestingly, we also found a significant *negative* effect of animacy, β = -1.94, se = 0.27, t(2068.99) = -7.18, p < .001. This effect indicates that manipulability ratings for inanimate objects are significantly *lower* than for animate objects, which might explain the inconsistent results concerning the effect of animacy on demonstrative use in Experiment 1 and Experiment 2.

However, animacy modulates the effects of harmfulness and size. The model displays a significant interaction between animacy and harmfulness, β = 2.06, se = 0.38, t(2068.97) = 5.41, p < .001, with differences in manipulability ratings between harmful and harmless objects being larger for inanimate objects compared to animate objects. Moreover, the interaction between animacy and size is significant, β = 3.35, se = 0.38, t(2068.97) = 8.78, p < .001. These two effects could be interpreted as further indirect evidence in favour of the sensory-functional hypothesis on the animate-inanimate distinction. Manipulability, a prototypical functional semantic feature, seems to be more distinctive of different types of inanimate objects than of types of animate objects.

Finally, the model displays a marginally significant interaction between harmfulness and size, β = -0.98, se = 0.38, t(2068.94) = -2.57, p < .05. A full summary of the model parameters and significance levels is provided in S3 Table. The model achieves a marginal R^2^ of 0.49, and a conditional R^2^ of 0.55. The relationship between manipulability scores and the three experimental variables is summarized in S4 Fig.

### Manipulability scores and demonstrative use

In order to assess the extent to which the effects of size and harmfulness are driven by manipulability rather than by residual variable-specific semantic information, we added the sum of manipulability scores as a parametric regressor to the model used to analyze the data from Experiment 2.

In this analysis we first fitted the original model used for Experiment 2 to the Danish data. In line with the cumulative analysis, the model reveals main effects of Size, β = 0.57, se = 0.13, z = 4.35, p < .001, and Harmfulness, β = 0.36, se = 0.13, z = 2.72, p < .01, as well as interactions between Animacy and Size, β = 0.79, se = 0.18, z = 4.43, p < .001, and Animacy and Harmfulness, β = 1.16, se = 0.18, z = 6.4, p < .001. The model achieves a marginal R^2^ of 0.169 and a conditional R^2^ of 0.39. We then compared the original model (df = 9, log-likelihood = -3656.5) to the extended one (df = 10, log-likelihood = -3645.1) using ANOVA. Adding the manipulability regressor significantly improved the model fit, χ^2^(1) = 22.73, log-likelihood difference = -11.4, p < .001. The explicit manipulability scores thus explain an additional part of the variance in the data. This suggests that, alongside the three dimensions tested in our experiments, additional functional features contributing to an object’s manipulability play a role in demonstrative choice.

The extended model displays a significant effect of manipulability, β = 0.07, se = 0.01, z = 4.78, p < .001, indicating that, the more manipulable the referent, the higher the proportion of proximal demonstrative used to refer to it. There was no significant effect of Animacy, β = 0.13, se = 0.14, z = 0.91, p >.05, nor a significant effect of Size, β = 0.27, se = 0.14, z = 1.85, p > .05. The effect of Harmfulness was marginally significant, β = 0.28, se = 0.13, z = 2.12, p < .05.

The absence of a main effect of size and the reduced reliability of the effect of Harmfulness indicate that manipulability explains away the majority of the variance previously accounted for by these variables. This provides further support to the claim that the effects of Size and Harmfulness were indeed due to these variables encoding distinctions in manual affordances. However, while the effect of Size is entirely explained away by manipulability, the residual effect of Harmfulness suggests that additional semantic information encoded by this dimension might influence for demonstrative choice. It could be hypothesized that the valence component intrinsic to the harmful / harmless distinction independently modulates demonstrative choice, a claim that remains to be addressed in future experiments.

As in the previous model, there were significant interactions between Animacy and Harmfulness, β = 1.02, se = 0.18, z = 5.56, p < .001, as well as between Animacy and Size, β = 0.57, se = 0.19, z = 3.1, p < .01. In the extended model, marginal R^2^ increases to 0.172 and conditional R^2^ increases to 0.394.

**Fig 5:**
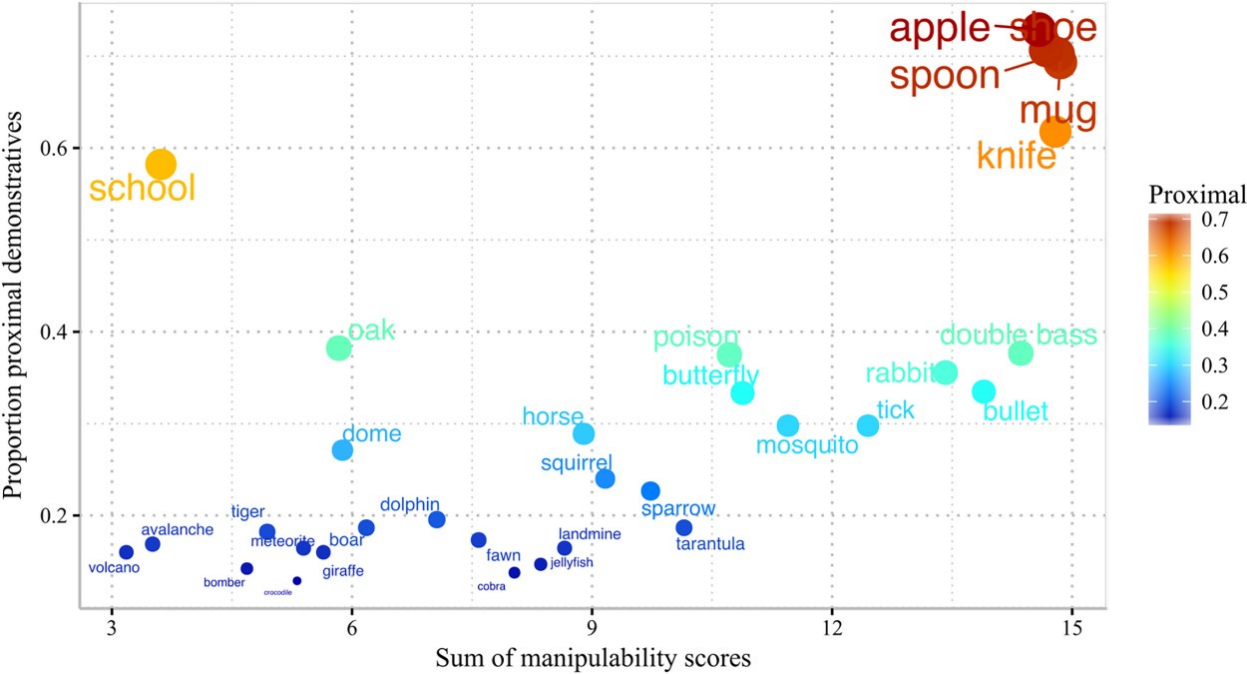
Proportion of proximal demonstratives as a function of manipulability. The proportion of proximal demonstratives used by participants in relation to specific words increases as a function of manipulability. Here, manipulability is obtained as the average (across participants) of the sum of the three manipulability ratings.

A full summary of the estimated parameters and their significance levels for both models is provided in S4 Table. The relationship between manipulability scores and the proportion of proximal demonstratives for each word is illustrated in Fig 5.

## Discussion

### Beyond the distance-based account

In our experiment, we investigated whether different types of objects, which varied in the degree to which they afford for manual interaction, consistently elicit different patterns of demonstrative use, regardless of the spatial context of utterance. Following our predictions, our results showed that speakers were more likely to use proximal demonstratives for objects more readily affording manipulation. Harmfulness and size of the referent affected participants’ choices of demonstrative forms across two experiments and three different languages, with participants being more likely to use proximal forms when presented with words denoting smaller compared to bigger referents, and harmless compared to harmful referents. As predicted, such effects were significantly stronger for inanimate referents, compared to animate beings.

However, contrary to our prediction, we only found evidence for a main effect of animacy in the first of two experiments. This might suggest that, contrary to our assumptions, the animate-inanimate distinction does not itself map onto a functional distinction between objects affording interaction to different extents, as suggested also by the analysis of post-hoc manipulability ratings. While animacy consistently influenced demonstrative use by modulating the effect of other functional dimensions, there is no conclusive evidence in favor of an independent preference for proximal demonstratives for inanimate objects, compared to animate beings.

Our results are consistent across the three languages tested in the experiment, which shows that the observed effects extend beyond patterns specific to a single language. We are nonetheless aware that the languages sampled in the current study are only representative of a restricted group within one language family, and we are not aiming at claiming cross-linguistic universality for such effects.

### Manual affordances and object semantics

Our studies provide a range of novel contributions to studies on demonstrative reference, which so far have almost exclusively focused on mapping the proximal/distal contrast onto spatial properties of the referents.

As our results indicate, spatial factors are likely to only account for part of the causal mechanisms underlying the usage profile of demonstratives. Dyadic demonstrative contrasts do not only encode the position of the referent relative to the speaker or the dyad, but additionally rely on semantic representations involving several functional dimensions along an object’s feature space. More specifically, functional properties of objects, i.e. the extent to which objects afford manual interaction, seem to be among the core factors shaping demonstrative use. However, while the link between demonstrative use and object semantics is a novel contribution to current research on spatial deixis, our results are also in continuity with existing literature. Indeed, although on a purely spatial basis, recent studies have converged on suggesting that the proximal/distal distinction is grounded in functional hand-centered representations of space and biomechanical affordances [23, 25–26]. In our experiments, we tested and expanded these hypotheses by targeting *non-spatial* dimensions that affect the extent to which a referent is available for manipulation. We focused on size and harmfulness as prototypical examples of functional properties that play a crucial role in defining an objects’ readiness for manual grasp, as well as on animacy as a factor which is thought to be intrinsically linked to the functional profile of objects.

Although size and harmfulness both play a role in the functional profile of referents, they reflect different ways in which manual affordances are relevant to demonstrative use. In the case of size, the difference between small and big referents is entirely determined by the physical and perceptual properties of a referent, and by purely biomechanical affordances. On the other hand, the effect of harmfulness projects the distinction to a deeper cognitive level, as it is likely to involve richer action representations, and arguably emotional components. In this respect, the effect of harmfulness could be interpreted as a more complex effect also involving object valence, that is of the degree to which manipulating an object is desirable, rather than merely physically possible.

### Demonstratives as a window onto semantic knowledge

Crucially, we also observed that preferences for proximal demonstratives for smaller over bigger objects, and for harmless over harmful objects were more pronounced for inanimate objects compared to animate objects.

This finding is in line with our predictions formulated along the line of a sensory-functional view on semantic knowledge. Sensory-functional theories are based on the claim that functional features are more prominent in semantic representations of inanimate objects compared to animate objects [38, 40–43]. As opposed to domain-specific, sensory-functional views explain the distinction between representations for inanimate and animate objects in terms of a difference in the degree to which functional and sensory features are involved in the representation of category tokens. Functional features are thought to be more prominent than sensory features in the representation of inanimate objects, while the relative importance of functional features is lower in the case of representations of animate beings. The core assumptions of sensory-functional theories were central to our predictions, and crucial to explain our main findings.

The central claims of the sensory-functional view on the animate-inanimate distinction are compatible with the observed effects of harmfulness and size. Though functional features are claimed to be more prominent in the case of inanimate objects, in a sensory-functional framework they are still thought to play a role in both the representation of animate beings and of inanimate objects. This leads to the prediction that within-category differences in harmfulness and size should affect patterns of demonstrative use both within animate beings and inanimate objects, which is reflected in the observed main effects of size and harmfulness.

A central tenet of sensory-functional theories is furthermore that functional features are more prominent for inanimate objects than for animate beings. This leads to the prediction that differences in affordance for grasp and manipulation should more heavily affect patterns of demonstrative use in the case of inanimate objects, than in that of animate beings. The interactions observed between animacy and harmfulness, and between animacy and size are in line with this hypothesis.

The congruency between our results and the sensory-functional hypothesis adds a further dimension to the significance of our results. As mentioned, the observed effects provide support to the idea that demonstrative reference is partly grounded in perceptual-motor affordances and biomechanical constraints. This is undoubtedly a crucial point in itself, as it brings to light the pivotal role of demonstratives as powerful interfaces between language, cognition and action. However, the observed relationship between demonstrative usage patterns and the animate-inanimate distinction contributes to the long-standing debate between domain-specific and modality-specific views on semantic knowledge by lending support to some of the predictions of sensory-functional approaches. While still not providing conclusive evidence, these results display the potential of using demonstratives to shed light onto core aspects in the organization of human semantic knowledge.

### Future directions: A comprehensive semantic analysis

In the present study, we selected a controlled set of words that varied along three target dimensions. As illustrated in the methods section, the selection process was tightly controlled, and the results hold even when accounting for item-specific patterns via a conservative random effects structure, which provides robust evidence for replicability of our effects. Still some variability in patterns of demonstrative choice can be observed both across languages and within levels of the experimental variables.

For example, while the effects of interest hold robustly across languages, the analyses displayed some cross-linguistic variation in the strength of the two-way interactions. As previously mentioned, these fluctuations might reflect false negatives, differences in sample size, or actual discrepancies in patterns of demonstrative use or word semantics.

**Fig 6:**
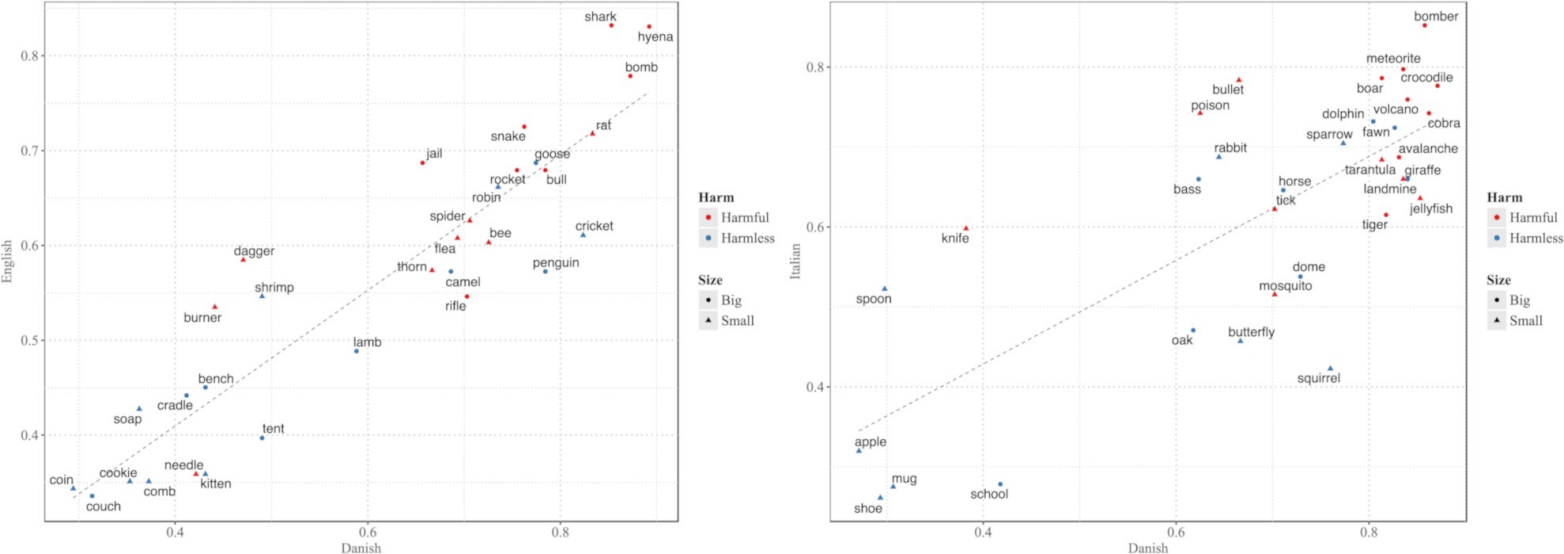
**Proportion of distal demonstratives for each stimulus word across languages for Experiment 1 (left) and Experiment 2 (right).** On the x axis the proportion of distal demonstratives for each word in Danish. On the y axis the proportion of distal demonstratives for each stimulus word in English for Experiment 1 and in Italian for Experiment 2. The diagonal line indicates the linear fit between the two. Words located closer to the fitted line display a similar use of demonstratives across languages, while words located further away from the fitted line elicited different use of demonstratives across languages. While there are no obvious patterns related to the experimental manipulations, demonstrative use for some particular words differs substantially across languages. This might reflect cross-cultural differences with respect to specific objects, i.e. more/less exposure to certain objects, or differences in the semantics of specific words across languages. The plot also displays within-category variability in demonstrative use consistent across languages.

Additionally, as displayed in Fig 6A and Fig 6B, a few specific words elicited a markedly different use of demonstratives across languages. It could be hypothesized that this is due to differences in the semantic profile of these words across languages, reflecting cross-cultural variation in exposure to certain referents. To provide a concrete example, specific animals might be present in certain countries but not in others, and customs or social practices involving the use of specific objects can vary across cultures, which could affect both familiarity with a certain referent and its perceived valence.

On the other hand, the plots also display some language-independent variability in the proportion of proximal demonstratives for individual tokens *within* levels of the experimental variables, with some words consistently showing higher proportions of proximal demonstratives compared to other items of the same category.

While, on the one hand, such patterns might reflect random fluctuations in the usage of demonstratives, it cannot be excluded that they reflect systematic variations due to further semantic factors that have not been explored in the present study.

Indeed, while in our experiments we found support for particular central semantic dimensions modulating the use of demonstratives, they are probably not exhaustive. A larger word sample and more fine-grained analyses of the semantic structure of the chosen stimulus set would help elucidating whether such variability can be accounted for in terms of other unpredicted semantic effects. Moreover, as reflected by the R^2^ estimates for the statistical models, a large part of the variance remains unexplained. Elucidating whether such variance is unsystematic or whether it reflects consistent effects of unexplored variables remains an open challenge for future work.

A range of methodologies could prove crucial in tackling these questions. For instance, the use of large semantic knowledge databases (e.g. [52]) would open up a wealth of possibilities. Such databases can be integrated with corpus data on the distributional profile of demonstratives or with experimental data, in order to better characterize the mapping between usage patterns of spatial demonstratives and the semantic profile of referents. It is to be noted, however, that at the moment the availability of such databases is only limited to a very small language sample, which therefore partly limits the possibility of drawing inferences on a cross-linguistic scale.

### Spatial context, biomechanics and object semantics: An integrated account

Taken together, our results show that spatial demonstratives are sensitive to functional properties of their referents, and they lend support to the hypothesis that the proximal/distal contrast is grounded in differences in affordances for manual interaction. While previous studies have focused on mapping demonstrative contrasts onto spatial properties of referents, our studies indicate that object semantics is a complementary factor in shaping the usage profile of spatial demonstratives.

However, in the present study we aimed at exploring the role of functional properties of referents independently of their spatial location, that is, without any spatial or perceptual context. Our results therefore cannot be used to generate any predictions on how object semantics and spatial properties interact, as it is likely to be the case in naturalistic contexts. For this purpose, new experimental paradigms will have to be used, where both the type of referent and its spatial properties are manipulated systematically.

The lack of perceptual context in this paradigm calls for a further remark. As no visual context was provided in the study, it might be objected that the effects observed rather reflect biases in visual imagery that would also partly be accounted for by as distance effect. For instance, it could be hypothesized that dangerous things tend to be mentally represented as further away compared to harmless objects, as a form of psychological distancing from undesirable objects. The same might hold for bigger objects, for instance those that could act as landmarks (such as buildings or natural entities), compared to smaller objects. Our paradigm does not directly allow us to test this possibility. This hypothesis, however, would only account for part of the effects (e.g. it would not explain the interactions), and it is hard to relate to the existing literature. Moreover, the notion of psychological distance in mental imagery would also likely reflect experiences and expectations about affordances for interaction, and the two hypotheses may thus reflect two sides of the same coin.

## Conclusions

Previous experimental work on demonstrative reference converges on showing that the distinction between proximal and distal spatial demonstratives might be grounded in functional properties of referents, such as their affordances for manual interaction. However, all studies so far have focused on mappings between the proximal/distal contrast and manual affordances in terms of the position of the referent relative to the speaker, to the conversational dyad, or to competing referents.

Our work aimed at investigating whether functional semantic properties of objects systematically influence speakers’ preferences for proximal or distal demonstrative forms, regardless of any spatial context.

Over two experiments, we presented speakers of English, Danish and Italian with words denoting animate and inanimate referents which differed in size (big/small) and harmfulness (harmful/harmless), and we asked them to match each word to either a proximal or a distal demonstrative form. Participants were more likely to use proximal demonstratives for harmless compared to harmful referents, and for small compared to big referents, that is for referents which are more likely to afford manual interaction. These effects provided robust evidence in favor of the hypothesis that the proximal/distal contrast is modulated by functional properties of objects, and specifically by their affordances for manual interaction.

Moreover, as predicted, such effects were stronger for inanimate objects, compared to animate referents. This is in line with the predictions of sensory-functional approaches to semantic knowledge, which claim that categorical distinctions between semantic representations of animate and inanimate beings can be mapped onto differences in the prominence of functional features over sensory features across the two categories.

Our study provides a two-fold contribution to the literature. On the one hand, our results provide further evidence for a grounding of spatial demonstratives onto manual affordances, with object semantics and biomechanical constraints being identified as complementary forces in shaping the usage profile of spatial demonstratives. On the other hand, they hint at the possibility of using demonstrative contrasts as proxies to investigate core aspects of the organization of human semantic knowledge.

## Supporting information

S1 Table Stimulus list for Experiment 1, all languages. (DOCX)

S2 Table Stimulus list for Experiment 2, Italian and Danish. (DOCX)

S3 Table Manipulability scores as a function of size, animacy and harmfulness, model summary. (DOCX)

S4 Table Demonstrative use as a function of experimental variables and manipulability scores for Danish data in Experiment 2. (DOCX)

S5 Table Overview of statistical model for Italian data from Experiment 1 without Soundness regressor. (DOCX)

S6 Table Overview of statistical model for Italian data from Experiment 1 including Soundness regressor. (DOCX)

S7 Table Overview of statistical model for parametric analysis. (DOCX)

S1 Fig Word frequencies for Experiment 1. Lemma occurrences per million words for each stimulus word in (A) Danish; (B) English; (C) Italian.
(TIF)

S2 Fig Distribution of word frequencies from Experiment 2. Lemma occurrences per million words for each stimulus word in (A) Danish; (B) Italian.
(TIF)

S3 Fig Overview of Italian data from Experiment 1. (TIF)

S4 Fig Manipulability scores for Experiment 2 as a function of size, harmfulness and animacy. (TIF)

S1 Appendix Analysis of Italian data from Experiment 1. (DOCX)

S2 Appendix Parametric analysis. (DOCX)
