# Supplementary material for: This shoe, that tiger: Semantic properties reflecting manual affordances of the referent modulate demonstrative use
Source: PLoS One. 2019 Jan 7;14(1):e0210333. doi: 10.1371/journal.pone.0210333 (PMC6322739; doi:10.1371/journal.pone.0210333)
Supplement: S1 Table — (DOCX) [file pone.0210333.s001.docx]

**S1 Table: Stimulus list for Experiment 1, all languages.**

Animate:

|  | Big | Small |
| --- | --- | --- |
| Harmful | Bull, shark, hyena, snake  Toro, squalo, iena, serpente  Tyr, haj, hyæne, slange | Bee, flea, spider, rat  Ape, pulce, ragno, ratto  Bi, loppe, edderkop, rotte |
| Harmless | Camel, goose, lamb, penguin  Cammello, oca, agnello, pinguino  Kamel, gås, lam, pingvin | Cricket, kitten, robin, shrimp  Grillo, gattino, pettirosso, gamberetto  Fårekylling, killing, rødhals, reje |

Inanimate:

|  | Big | Small |
| --- | --- | --- |
| Harmful | Bomb, jail, rocket, rifle  Bomba, prigione, razzo, fucile  Bombe, fængsel, missil, riffel | Burner, dagger, needle, thorn  Bruciatore, pugnale, ago, spina  Blus, dolk, nål, torn, |
| Harmless | Bench, couch, cradle, tent  Panca, divano, culla, tenda  Bænk, sofa, vugge, telt | Coin, comb, cookie, soap  Moneta, pettine, biscotto, sapone  Mønt, kam, småkage, sæbe |

Fillers:

| Deal, dawn, hurry, loss, quarrel, rise, rest, stress  Accordo, ascesa, fretta, perdita, alba, riposo, lite, stress  Aftale, fremgang, hastværk, tab, daggry, pause, skænderi, stress |
| --- |
