# Supplementary material for: This shoe, that tiger: Semantic properties reflecting manual affordances of the referent modulate demonstrative use
Source: PLoS One. 2019 Jan 7;14(1):e0210333. doi: 10.1371/journal.pone.0210333 (PMC6322739; doi:10.1371/journal.pone.0210333)
Supplement: S2 Table — (DOCX) [file pone.0210333.s002.docx]

**S2 Table: Stimulus list for Experiment 2, Italian and Danish.**

Animate:

|  | Big | Small |
| --- | --- | --- |
| Harmful | Tigre, cobra, coccodrillo, cinghiale  Tiger, kobra, krokodille, vildsvin  *(Tiger, cobra, crocodile, boar)* | Zanzara, medusa, tarantola, zecca  Myg, giftgople, fugleedderkop, skovflåt  *(Mosquito, jellyfish, tarantula, tick)* |
| Harmless | Giraffa, cavallo, delfino, cerbiatto  Giraf, hest, delfin, rådyr  *(Giraffe, horse, dolphin, fawn)* | Coniglio, farfalla, scoiattolo, passero  Kanin, sommerfugl, egern, spurv  *(Rabbit, butterfly, squirrel, sparrow)* |

Inanimate:

|  | Big | Small |
| --- | --- | --- |
| Harmful | Meteorite, valanga, vulcano, bombardiere  Meteor, lavine, vulkan, bombefly  *(Meteorite, avalanche, volcano, bomber)* | Mina, veleno, proiettile, coltello  Landmine, gift, patron, kniv  *(Mine, poison, bullet, knife)* |
| Harmless | Scuola, quercia, contrabbasso, cupola  Skole, egetræ, kontrabas, kuppel  *(School, oak, double-bass, dome)* | Tazza, scarpa, cucchiaio, mela  Krus, sko, ske, æble  *(Mug, shoe, spoon, apple)* |

Fillers:

| Cambiamento, delusione, pace, promozione, scomparsa, sconfitta, sorpresa, vergogna  Forandring, skuffelse, fred, forfremmelse, forsvinden, nederlag, overraskelse, skam  *(Change, disappointment, peace, promotion, disappearance, defeat, surprise, shame)* |
| --- |
