# Supplementary material for: This shoe, that tiger: Semantic properties reflecting manual affordances of the referent modulate demonstrative use
Source: PLoS One. 2019 Jan 7;14(1):e0210333. doi: 10.1371/journal.pone.0210333 (PMC6322739; doi:10.1371/journal.pone.0210333)
Supplement: S3 Table — (DOCX) [file pone.0210333.s003.docx]

**S3 Table. Manipulability scores as a function of size, animacy and harmfulness, model summary**

|  | **Beta** | **SE** | | **df** | **t** | **95% CI**  **lower** | **95% CI**  **upper** | **p** |
| --- | --- | --- | --- | --- | --- | --- | --- | --- |
| (Intercept) | 6,12 | | 0,23 | 359,1491 | 26,22 | 5,67 | 6,57 | <.001*** |
| Harm | 1,18 | | 0,27 | 2068,947 | 4,37 | 0,65 | 1,71 | <.001*** |
| Size | 4,49 | | 0,27 | 2068,947 | 16,64 | 3,96 | 5,02 | <.001*** |
| Animate | -1,94 | | 0,27 | 2068,998 | -7,18 | -2,47 | -1,41 | <.001*** |
| Harm x Size | -0,98 | | 0,38 | 2068,947 | -2,57 | -1,72 | -0,24 | <.05 * |
| Harm x Animate | 2,06 | | 0,38 | 2068,972 | 5,41 | 1,32 | 2,8 | <.001*** |
| Size x Animate | 3,35 | | 0,38 | 2068,972 | 8,78 | 2,61 | 4,09 | <.001*** |
| Harm x Size x Animate | 0,46 | | 0,54 | 2068,96 | 0,84 | -0,6 | 1,52 | n.s. |
