# Supplementary material for: This shoe, that tiger: Semantic properties reflecting manual affordances of the referent modulate demonstrative use
Source: PLoS One. 2019 Jan 7;14(1):e0210333. doi: 10.1371/journal.pone.0210333 (PMC6322739; doi:10.1371/journal.pone.0210333)
Supplement: S4 Table — (DOCX) [file pone.0210333.s004.docx]

**S4 Table. Demonstrative use as a function of experimental variables and manipulability scores for Danish data in Experiment 2**

A. Without manipulability regressor

|  | **Beta** | **SE** | **z** | **95% CI**  **lower** | **95% CI**  **upper** | **Odds**  **Ratio** | **p** |
| --- | --- | --- | --- | --- | --- | --- | --- |
| (Intercept) | -2.00 | 0,12 | -16,17 | -2,24 | -1,76 | 0,14 | <.001*** |
| Animate | 0 | 0,14 | 0 | -0,27 | 0,27 | 1 | n.s. |
| Size | 0,57 | 0,13 | 4,35 | 0,32 | 0,82 | 1,77 | <.001*** |
| Harm | 0,36 | 0,13 | 2,72 | 0,11 | 0,61 | 1,43 | <.01 ** |
| Animate x Size | 0,79 | 0,18 | 4,43 | 0,44 | 1,14 | 2,2 | <.001*** |
| Animate x Harm | 1,16 | 0,18 | 6,4 | 0,81 | 1,51 | 3,19 | <.001*** |
| Size x Harm | -0,02 | 0,18 | -0,13 | -0,37 | 0,33 | 0,98 | n.s. |
| Animate x Size x Harm | 0,22 | 0,24 | 0,92 | -0,25 | 0,69 | 1,25 | n.s. |

B. Extended (including manipulability)

|  | **Beta** | **SE** | **z** | **95% CI lower** | **95% CI upper** | **Odds Ratio** | **p** |
| --- | --- | --- | --- | --- | --- | --- | --- |
| (Intercept) | -2,41 | 0,15 | -15,95 | -2,7 | -2,12 | 0,09 | <.001*** |
| Manipulability | 0,07 | 0,01 | 4,78 | 0,05 | 0,09 | 1,07 | <.001*** |
| Animate | 0,13 | 0,14 | 0,91 | -0,14 | 0,4 | 1,14 | n.s. |
| Size | 0,27 | 0,14 | 1,85 | 0 | 0,54 | 1,31 | n.s. |
| Harm | 0,28 | 0,13 | 2,12 | 0,03 | 0,53 | 1,32 | <.05 * |
| Animate x Size | 0,57 | 0,19 | 3,1 | 0,2 | 0,94 | 1,77 | <.01 ** |
| Animate x Harm | 1,02 | 0,18 | 5,56 | 0,67 | 1,37 | 2,77 | <.001*** |
| Size x Harm | 0,04 | 0,18 | 0,25 | -0,31 | 0,39 | 1,04 | n.s. |
| Animate x Size x Harm | 0,2 | 0,24 | 0,81 | -0,27 | 0,67 | 1,22 | n.s. |
