# Supplementary material for: This shoe, that tiger: Semantic properties reflecting manual affordances of the referent modulate demonstrative use
Source: PLoS One. 2019 Jan 7;14(1):e0210333. doi: 10.1371/journal.pone.0210333 (PMC6322739; doi:10.1371/journal.pone.0210333)
Supplement: S5 Table — (DOCX) [file pone.0210333.s005.docx]

**S5 Table. Overview of statistical model for Italian data from Experiment 1 without Soundness regressor**

|  | **Beta** | **SE** | **z** | **95% CI**  **lower** | **95% CI**  **upper** | **Odds**  **Ratio** | **p** |
| --- | --- | --- | --- | --- | --- | --- | --- |
| (Intercept) | -0,09 | 0,11 | -0,79 | -0,31 | 0,13 | 0,91 | n.s. |
| Animate | 0,02 | 0,13 | 0,13 | -0,23 | 0,27 | 1,02 | n.s. |
| Size | 0,3 | 0,13 | 2,3 | 0,05 | 0,55 | 1,35 | <.05 * |
| Harm | 0,45 | 0,13 | 3,42 | 0,2 | 0,7 | 1,57 | <.001*** |
| Animate x Size | 0,25 | 0,19 | 1,32 | -0,12 | 0,62 | 1,28 | n.s. |
| Animate x Harm | -0,07 | 0,19 | -0,38 | -0,44 | 0,3 | 0,93 | n.s. |
| Size x Harm | 0,19 | 0,19 | 1,02 | -0,18 | 0,56 | 1,21 | n.s. |
| Animate x Size x Harm | 0,29 | 0,27 | 1,05 | -0,24 | 0,82 | 1,34 | n.s. |
