# Supplementary material for: This shoe, that tiger: Semantic properties reflecting manual affordances of the referent modulate demonstrative use
Source: PLoS One. 2019 Jan 7;14(1):e0210333. doi: 10.1371/journal.pone.0210333 (PMC6322739; doi:10.1371/journal.pone.0210333)
Supplement: S6 Table — (DOCX) [file pone.0210333.s006.docx]

**S6 Table. Overview of statistical model for Italian data from Experiment 1 including Soundness regressor.**

|  | **Beta** | **SE** | **z** | **95% CI**  **lower** | **95% CI**  **upper** | **Odds**  **Ratio** | **p** |
| --- | --- | --- | --- | --- | --- | --- | --- |
| (Intercept) | -0,78 | 0,14 | -5,55 | -1,05 | -0,51 | 0,46 | <.001*** |
| Animate | -0,06 | 0,16 | -0,34 | -0,37 | 0,25 | 0,94 | n.s. |
| Size | 0,72 | 0,16 | 4,5 | 0,41 | 1,03 | 2,05 | <.001*** |
| Harm | 0,91 | 0,15 | 5,95 | 0,62 | 1,2 | 2,48 | <.001*** |
| Soundness | 1,37 | 0,14 | 9,43 | 1,1 | 1,64 | 3,94 | <.001*** |
| Animate x Size | 0,44 | 0,2 | 2,21 | 0,05 | 0,83 | 1,55 | <.05 * |
| Animate x Harm | -0,03 | 0,2 | -0,15 | -0,42 | 0,36 | 0,97 | n.s. |
| Size x Harm | -0,22 | 0,24 | -0,89 | -0,69 | 0,25 | 0,8 | n.s. |
| Animate x Soundness | 0,15 | 0,17 | 0,88 | -0,18 | 0,48 | 1,16 | n.s. |
| Harm x Soundness | -0,33 | 0,18 | -1,86 | -0,68 | 0,02 | 0,72 | n.s. |
| Size x Soundness | -0,8 | 0,17 | -4,78 | -1,13 | -0,47 | 0,45 | <.001*** |
| Animate x Size x Harm | 0,09 | 0,32 | 0,28 | -0,54 | 0,72 | 1,09 | n.s. |
