# Supplementary material for: This shoe, that tiger: Semantic properties reflecting manual affordances of the referent modulate demonstrative use
Source: PLoS One. 2019 Jan 7;14(1):e0210333. doi: 10.1371/journal.pone.0210333 (PMC6322739; doi:10.1371/journal.pone.0210333)
Supplement: S7 Table — (DOCX) [file pone.0210333.s007.docx]

**S7 Table. Overview of statistical model for parametric analysis.**

|  | **Beta** | **SE** | **z** | **95% CI**  **lower** | **95% CI**  **upper** | **Odds**  **Ratio** | **p** |
| --- | --- | --- | --- | --- | --- | --- | --- |
| (Intercept) | -0,16 | 0,09 | -1,89 | -0,34 | 0,02 | 0,85 | n.s. |
| Animate | 1,76 | 0,1 | 17,19 | 1,56 | 1,96 | 5,81 | <.001*** |
| SizeScore | -0,08 | 0,02 | -5,22 | -0,12 | -0,04 | 0,92 | <.001*** |
| HarmScore | -0,19 | 0,02 | -7,96 | -0,23 | -0,15 | 0,83 | <.001*** |
| Animate x SizeScore | -0,13 | 0,02 | -5,96 | -0,17 | -0,09 | 0,88 | <.001*** |
| Animate x HarmScore | -0,26 | 0,03 | -8,88 | -0,32 | -0,2 | 0,77 | <.001*** |
