# Supplementary figures and images for: This shoe, that tiger: Semantic properties reflecting manual affordances of the referent modulate demonstrative use
Source: PLoS One. 2019 Jan 7;14(1):e0210333. doi: 10.1371/journal.pone.0210333 (PMC6322739; doi:10.1371/journal.pone.0210333)

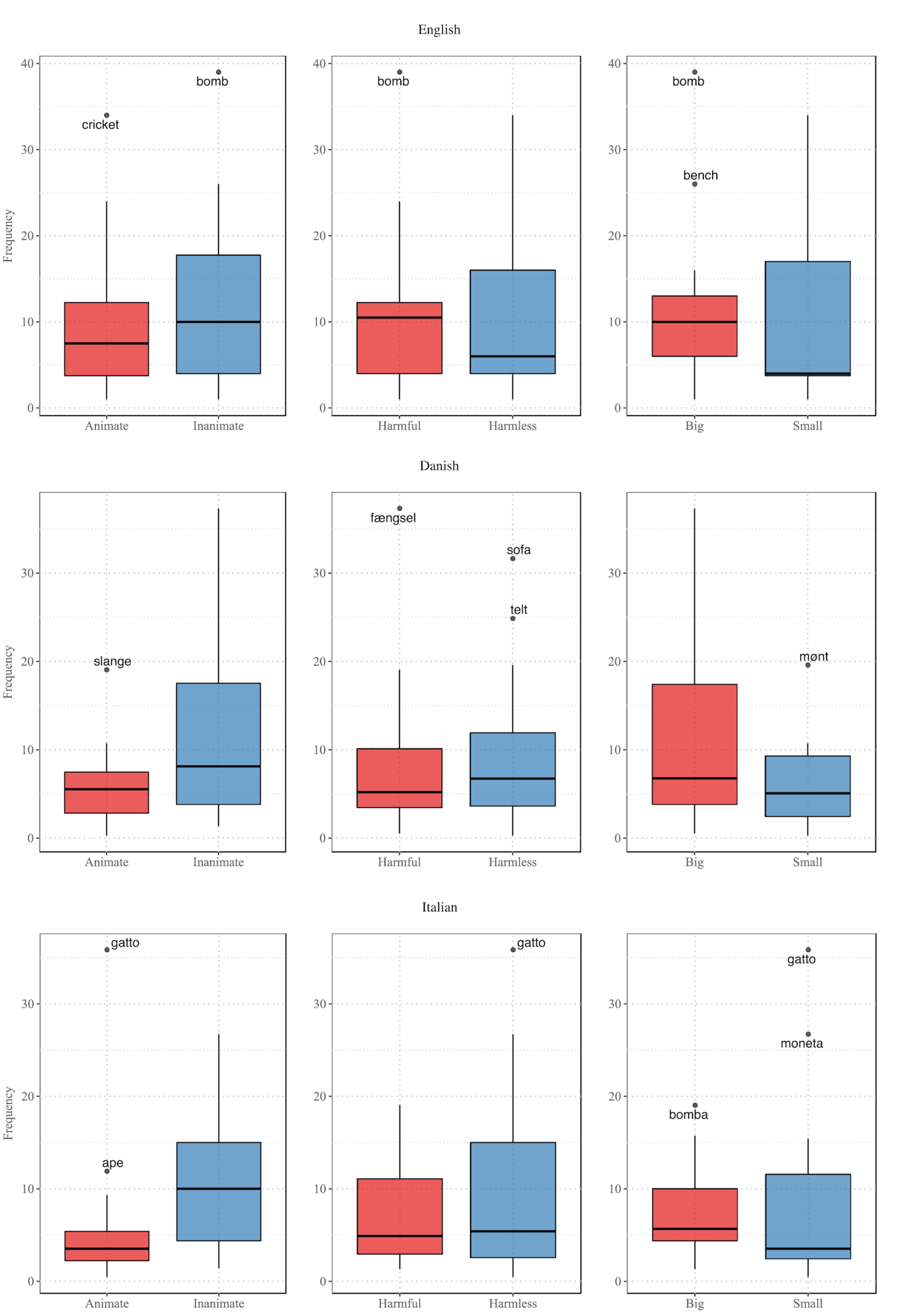

Supplement: S1 Fig — Lemma occurrences per million words for each stimulus word in (A) Danish; (B) English; (C) Italian. (TIF) [file pone.0210333.s008.tif]

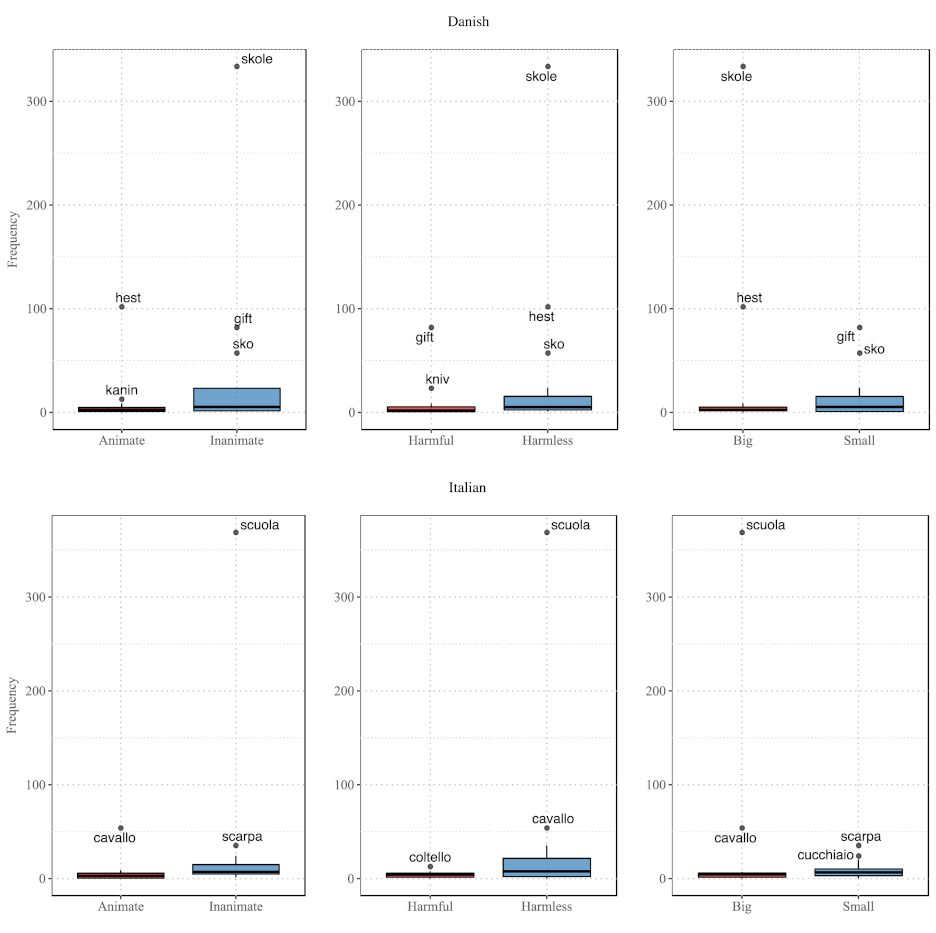

Supplement: S2 Fig — Lemma occurrences per million words for each stimulus word in (A) Danish; (B) Italian. (TIF) [file pone.0210333.s009.tif]

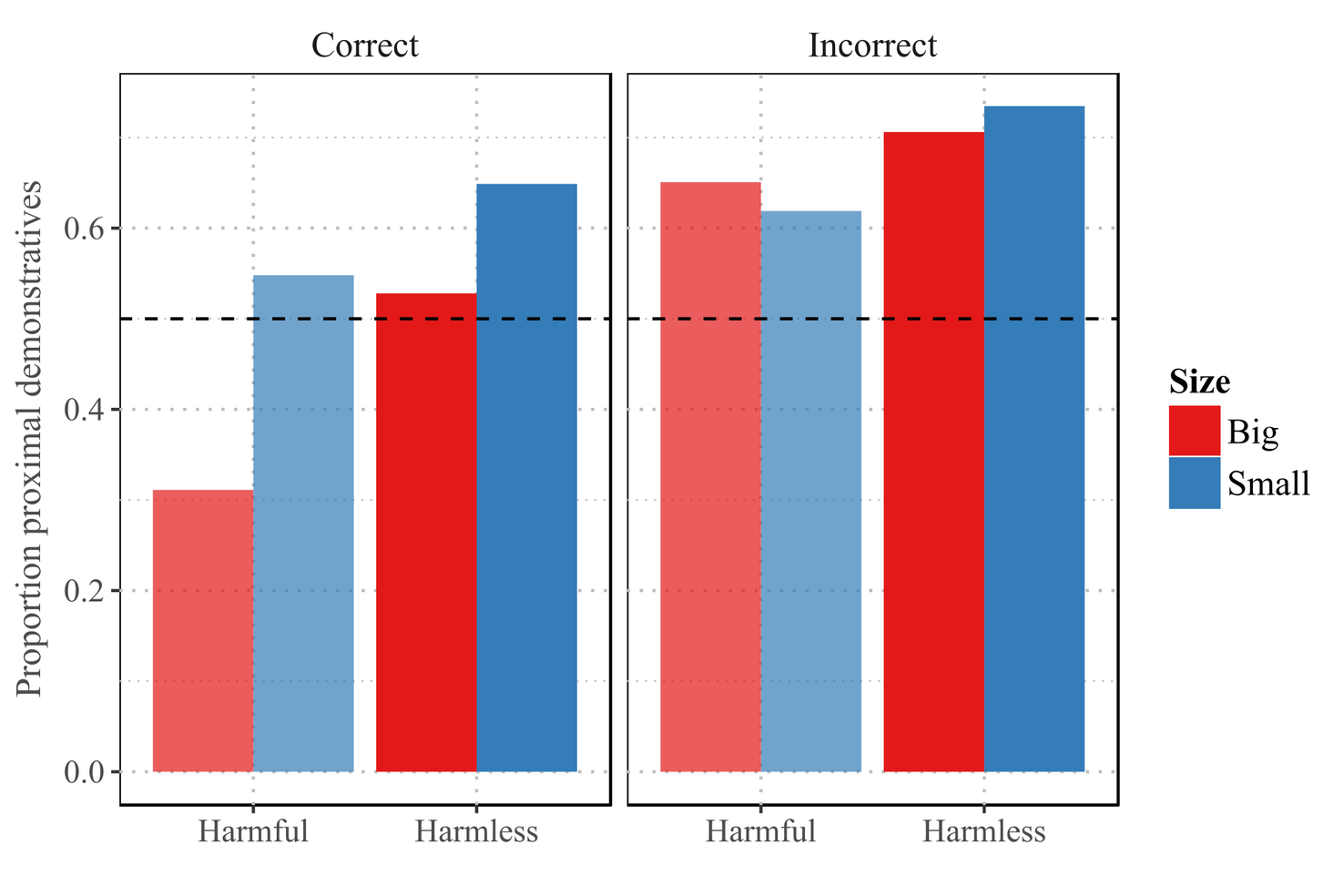

Supplement: S3 Fig — (TIF) [file pone.0210333.s010.tif]

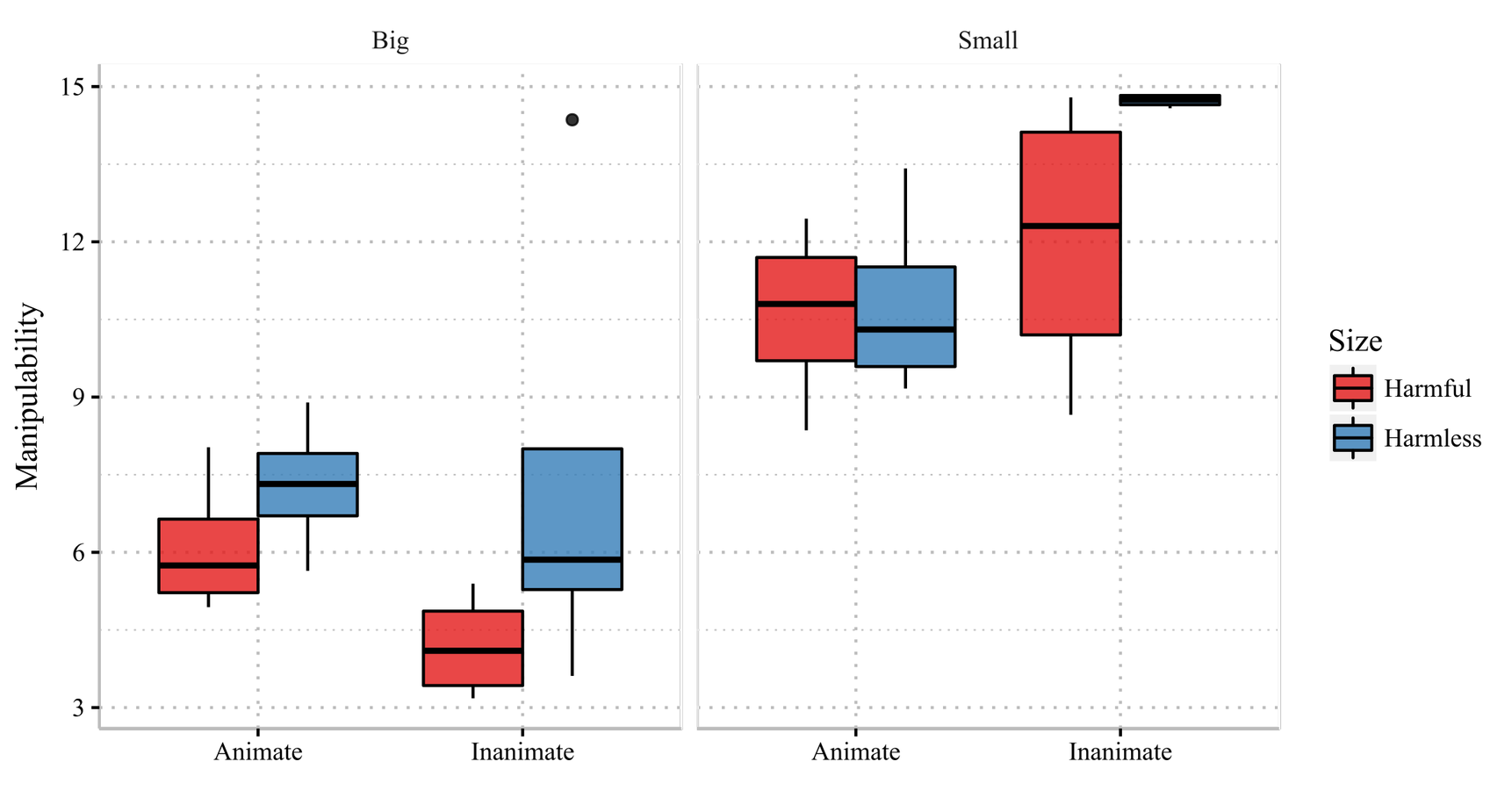

Supplement: S4 Fig — (TIF) [file pone.0210333.s011.tif]
