# Supplementary material for: This shoe, that tiger: Semantic properties reflecting manual affordances of the referent modulate demonstrative use
Source: PLoS One. 2019 Jan 7;14(1):e0210333. doi: 10.1371/journal.pone.0210333 (PMC6322739; doi:10.1371/journal.pone.0210333)
Supplement: S1 Appendix — (DOCX) [file pone.0210333.s012.docx]

**S1 Appendix. Analysis of Italian data from Experiment 1.**

As observed in the Methods section, a potential confound was present in the Italian experiment, as the demonstrative forms presented in order to make the experiment comparable across languages, were not always the most appropriate from a grammatical point of view. In some cases, which have been listed in detail in the methods section, the distal form *quel* would have been the most correct form, instead of the form *quello* which was uniformly presented all throughout the experiment. We therefore analyzed the Italian data from Experiment 1 separately, in order to detect whether such confound could have significantly impacted the results.

First, we fitted a mixed-effects logistic regression model including animacy, harmfulness and size, as well as all the interactions, in the fixed effects structure, and participants as random effects (intercept only). These data revealed a strong main effect of Harmfulness, β = 0.45, se = 0.13, z = 3.42, p < 0.001 in the predicted direction, as well as a main effect of Size, β = 0.3, se = 0.13, z = 2.3, p < 0.05. The model achieves a marginal R^2^ of 0.05, and a conditional R^2^ of 0.19. Details of the model are reported in S5 Table.

Then, we fitted a model including an additional binary regressor coding for whether the combination between the distal demonstrative and the noun was grammatically correct.

In cases coded as “incorrect”, i.e. cases where the masculine distal form *quel* would have been the most correct forms, it could be expected that participants displayed a tendency to use proximal demonstratives. We compared this model (df = 13, log-likelihood = -2632.9) to the simpler model (df = 9, log-likelihood = -2542.0) used above. The former explained significantly more variance, χ^2^(4) = 181.79, p < .001, thus suggesting that the introduction of the covariate coding for the grammatical soundness of the distal demonstratives improved the model fit.

Likelihood-ratio tests for the model revealed a significant main effect of this regressor, β = 1.37, se = 0.14, z = 9.34, p < .001, showing that the proportion of proximal demonstratives was indeed higher when the distal demonstrative was not entirely grammatically sound (S3 Fig).

Reflecting corpus frequency data, the extended model revealed an overall preference for distal demonstratives, β = -0.78, se = 0.14, z = -5.55, p < .001. There was a main effect of Harmfulness, β = 0.91, se = 0.15, z = 5.95, p < .001, and a main effect of Size, β = 0.72, se = 0.16, z = 4.5, p < .001, both in the predicted direction. Moreover, there was a significant interaction between Animacy and Size, β = 0.44 se = 0.2, z = 2.2, p < .05, with the effect of Size being stronger for inanimate objects, compared to animate beings. A significant interaction between Size and Soundness, β = -0.8, se = 0.17, z = -4.78, p < .001, revealed that the effect of Size was smaller in those cases were the distal demonstrative was not grammatically correct. No other effects reached statistical significance.

While the main effects from the Italian data further support effects observed in English and Danish, the latter effect is difficult interpretability, and makes it more challenging to draw any inferences on the hypothesis of animacy as modulator of the size and harmfulness hypothesized in the introduction.

The extended model displays a marginal R^2^ of 0.1, and a conditional R^2^ of 0.24. Details on the statistical model are reported in S6 Table.
