# Supplementary material for: This shoe, that tiger: Semantic properties reflecting manual affordances of the referent modulate demonstrative use
Source: PLoS One. 2019 Jan 7;14(1):e0210333. doi: 10.1371/journal.pone.0210333 (PMC6322739; doi:10.1371/journal.pone.0210333)
Supplement: S2 Appendix — (DOCX) [file pone.0210333.s013.docx]

**S2 Appendix. Parametric Analysis.**

In order to make sure that the effects observed in the logistic regression analyses using binary predictors are not artefacts of the categorization of words into binary distinctions, we conducted a parametric version of the cumulative analysis. We used mixed-effect logistic regression on data from Experiment 1 and 2, excluding the Italian dataset from Experiment 1 due to the above mentioned confounds. The fixed effects structure included size and harmfulness scores on a 1 to 5 scale, the binary predictor for animacy, and the interactions between animacy and size and animacy and harmfulness as fixed effects. The random effects structure included a random intercept for each participant, and the demonstrative used at each trial was the binary outcome variable.

There were significant main effects of harmfulness, β = -0.19, se = 0.02, z = -7.96, p < .001, of size, β = -0.08, se = 0.02, z = -5.22, p < .001 and of animacy, β = 1.75, se = 0.1, z = 17.19, p < .001. The interaction between animacy and harmfulness was significant, β =-0.26, se = 0.03, z = -8.88, p < .001, as well as the interaction between animacy and size, β = -0.12, se = 0.02, z = -5.96, p < .001.

The model achieves a marginal R^2^ of 0.082 and a conditional R^2^ of 0.245. Details on the statistical model are reported in S7 Table.
